# Synergizing hypomethylating agents with off-the-shelf CD70-targeted chimeric antigen receptor-engineered natural killer T cells for the treatment of acute myeloid leukemia

**DOI:** 10.1038/s41375-026-02930-5

**Published:** 2026-03-26

**Authors:** Yan-Ruide Li, Xinyuan Shen, Yuning Chen, Yichen Zhu, Jie Huang, Caspian Oliai, Lili Yang

**Affiliations:** 1 Department of Microbiology, Immunology & Molecular Genetics, University of California, Los Angeles, CA USA; 2 Department of Bioengineering, University of California, Los Angeles, CA USA; 3 Division of Hematology-Oncology, Department of Medicine, David Geffen School of Medicine, University of California, Los Angeles, CA USA; 4 Jonsson Comprehensive Cancer Center, David Geffen School of Medicine, University of California, Los Angeles, CA USA; 5 Eli and Edythe Broad Center of Regenerative Medicine and Stem Cell Research, University of California, Los Angeles, CA USA; 6 Molecular Biology Institute, University of California, Los Angeles, CA USA; 7 Parker Institute for Cancer Immunotherapy, University of California, Los Angeles, CA USA; 8 Goodman-Luskin Microbiome Center, University of California, Los Angeles, CA USA

**Keywords:** Immunotherapy, Cancer immunotherapy

## Abstract

Acute myeloid leukemia (AML) is an aggressive hematologic malignancy characterized by the uncontrolled expansion of undifferentiated myeloid precursors in the bone marrow. Hypomethylating agents (HMAs) such as azacitidine and decitabine can reverse abnormal DNA methylation, promote leukemic cell differentiation, and enhance immune recognition, yet relapse and therapeutic resistance remain major challenges. In this study, we found that long-term, low-dose HMA treatment upregulated CD70, NK receptor ligands, and CD1d on AML tumor cells, rendering them more susceptible to chimeric antigen receptor (CAR)-engineered invariant natural killer T (CAR-NKT) cell-mediated cytotoxicity. To exploit these features, we generated two types of CD70-targeting CAR-NKT cells: cord blood hematopoietic stem and progenitor cell (HSPC)-derived allogeneic CAR70-NKT (^Allo^CAR70-NKT) cells and peripheral blood mononuclear cell (PBMC)-derived CAR70-NKT (^PBMC^CAR70-NKT) cells. Both CAR70-NKT cell products exhibited potent cytotoxicity against AML cells and synergized with HMAs, while ^Allo^CAR70-NKT cells demonstrated superior antitumor efficacy, multi-target recognition, and sustained expansion. In multiple xenograft models, ^Allo^CAR70-NKT cells effectively killed AML tumors without inducing graft-versus-host disease, cytokine release syndrome, or long-term organ toxicity. These findings highlight ^Allo^CAR70-NKT cells as a safe and powerful off-the-shelf immunotherapy that can synergize with HMAs to improve treatment outcomes for patients with AML.

## Introduction

Acute myeloid leukemia (AML) is an aggressive hematologic malignancy characterized by the clonal proliferation and differentiation blockade of myeloid precursors in the bone marrow [1–3]. Despite advances in therapeutic options, the overall survival for older adults remains dismal, with a median overall survival of only 6-11 months [3–5]. Hypomethylating agents (HMAs), including azacitidine and decitabine, represent a lower-intensity therapeutic approach that can reverse aberrant DNA methylation and reprogram leukemic cells toward differentiation and immune recognition. Over the past two decades, these first-generation HMAs have become a mainstay in the treatment of AML and myelodysplastic syndromes, particularly in elderly or medically unfit patients [6–8]. However, their clinical efficacy remains limited by incomplete remission rates and relapse, underscoring the need for combinatorial strategies that can enhance anti-leukemic immunity while maintaining tolerability.

Various strategies have been explored to potentiate the therapeutic efficacy of HMAs through rational combination approaches. Notably, leukemia stem cells (LSCs) upregulate CD70 in response to HMA treatment, leading to enhanced CD70/CD27 signaling that promotes leukemic cell survival and immune evasion [9–11]. Therapeutically, blockade of this axis has shown promise, as demonstrated in clinical trials in previously untreated older patients with AML in which a single priming dose of cusatuzumab, a human anti-CD70 monoclonal antibody, followed by azacitidine combination therapy yielded high response rates with a favorable safety profile [10]. Beyond CD70, HMAs also induce the expression of other immune-regulatory and tumor-associated molecules on AML blasts, including CD80, CD86, PD-L1, CD1d, and NKG2D ligands, which may enhance immune recognition and render leukemic cells more susceptible to immune-mediated clearance [12–16]. Therefore, targeting HMA-induced molecular and immunologic vulnerabilities represents a promising strategy to fully leverage epigenetic therapy in AML.

Recently, we developed a clinically guided culture method to generate allogeneic CD70-targeting chimeric antigen receptor (CAR)-engineered invariant natural killer T (NKT) cells (denoted as ^Allo^CAR70-NKT cells) [17, 18]. By engineering the NKT T cell receptor (TCR) into human cord blood-derived CD34⁺ hematopoietic stem and progenitor cells (HSPCs) and directing their ex vivo differentiation, we successfully produced functional ^Allo^CAR70-NKT cells with potent cytotoxicity against CD70⁺ tumor cells [18]. These cells also demonstrated robust killing of CD70⁻ tumors through TCR- and natural killer receptor (NKR)-mediated mechanisms, highlighting their multi-modal antitumor activity [18]. Given that HMA treatment can upregulate CD70, CD1d (recognized by the NKT TCR), and NKR ligands (such as ULBPs and MICA/B) on AML blasts [10, 12, 16], combining HMAs with ^Allo^CAR70-NKT therapy may produce a synergistic antileukemic effect by enhancing target recognition and immune engagement.

In this study, we systematically investigate the therapeutic synergy between HMAs and CAR70-NKT cell therapy for AML. We perform a comprehensive side-by-side comparison of human HSPC-derived and peripheral blood mononuclear cell (PBMC)-derived CAR70-NKT cell products, evaluating their manufacturing, phenotype, and functionality. Furthermore, we characterize the HMA-induced upregulation of tumor antigens on AML blasts and assess how these molecular alterations enhance the AML susceptibility to CAR70-NKT cell-mediated cytotoxicity. We also investigate the safety profile of CAR70-NKT cells, including potential graft-versus-host disease (GvHD), cytokine release syndrome (CRS), and long-term organ toxicity. Collectively, our findings establish a translational foundation for the development of an off-the-shelf, safe, and efficacious allogeneic CAR70-NKT cell therapy for AML, with the potential to synergize with epigenetic modulation to achieve durable anti-leukemic responses.

## Materials and methods

Detailed protocols for Media and reagents, Mice, Antibodies and flow cytometry, Enzyme-linked immunosorbent cytokine assays (ELISAs), Histology analysis, and Statistics can be found in the Supplementary methods.

### Study approval

Animal studies were conducted under protocols approved by the UCLA Division of Laboratory Animal Medicine. Healthy donor PBMCs were obtained from the UCLA/CFAR Virology Core Laboratory and HemaCare under informed consent and in compliance with federal and state regulations; no identifying information was provided.

### Lentiviral vectors

A parental lentivector, pMNDW, was utilized to construct the lentiviral vectors employed in this study [19, 20]. The 2 A sequences derived from foot-and-mouth disease virus (F2A), porcine teschovirus-1 (P2A), and thosea asigna virus (T2A) were used to link the inserted genes to achieve co-expression. The Lenti/iNKT-CAR70-IL-15 vector was constructed by inserting into the pMNDW parental vector a synthetic tetracistronic gene encoding human iNKT TCRα-F2A-iNKT TCRβ-P2A-CAR70-T2A-IL-15 (CAR70 indicates a CD70-directed CAR, and IL-15 indicates the secreted form of human IL-15). The Lenti/CAR70 vector was constructed by inserting into the pMNDW parental vector a synthetic gene encoding CAR70. The Lenti/CAR70-IL-15 vector was constructed by inserting into the pMNDW parental vector a synthetic bicistronic gene encoding CAR70-F2A-IL-15. The Lenti/iNKT vector was constructed by inserting into the pMNDW parental vector a synthetic bicistronic gene encoding iNKT TCRα-F2A-iNKT TCRβ. The Lenti/CAR70-EGFP vector was constructed by inserting into the pMNDW parental vector a synthetic bicistronic gene encoding CAR70-F2A-EGFP. The Lenti/FG vector was constructed by inserting into pMNDW parental vector a synthetic bicistronic gene encoding Fluc-P2A-EGFP. The Lenti/CD70 vector was constructed by inserting into pMNDW parental vector a synthetic gene encoding human CD70. Synthetic gene fragments were sourced from GenScript (Piscataway, NJ, USA) and IDT (Coralville, IA, USA). Lentiviral particles were generated utilizing HEK 293 T cells by employing a standardized transfection procedure with the Trans-IT-Lenti Transfection Reagent (Mirus Bio) [19, 20]. Subsequently, a concentration protocol was applied using Amicon TM Ultra Centrifugal Filter Units in accordance with the manufacturer’s specifications (MilliporeSigma).

### Stable cell lines

Human acute monocytic leukemia cell line THP1 (cat. no. TIB-202), KG1 (cat. no. CCL-246), and acute promyelocytic leukemia cell line HL60 (cat. no. CCL-240) were purchased from the American Type Culture Collection (ATCC). To establish stable tumor cell lines that overexpress both firefly luciferase and enhanced green fluorescent protein dual reporters (FG), the tumor cell lines were transduced with lentiviral vectors carrying the specific genes of interest (i.e., Lenti/FG). Flow cytometry sorting was performed to isolate the GFP^+^ cells 72 h after lentiviral transduction to generate stable cell lines (i.e., THP1-FG, KG1-FG, and HL60-FG). The artificial antigen presenting cell line (aAPC) was generated by engineering the K562 human chronic myelogenous leukemia cell line (ATCC, cat. no. CCL-243) to overexpress human CD80/CD83/CD86/41BBL co-stimulatory receptors. The aAPC-CD70 cell lines were generated by further engineering the parental aAPC line to overexpress human CD70. All cell lines used in this study were authenticated by short tandem repeat (STR) profiling and were confirmed to be free of mycoplasma contamination.

### Human CD34^+^ hematopoietic stem and progenitor cells (HSPCs), and peripheral blood mononuclear cells (PBMCs)

Purified human CD34^+^ HSPCs derived from cord blood (CB) were purchased from HemaCare. Healthy donor PBMCs were provided by the UCLA/CFAR Virology Core Laboratory without identification information under federal and state regulations. Upon receipt, both HSPCs and PBMCs were promptly aliquoted and cryopreserved in liquid nitrogen for subsequent experimental use.

### Generation of HSPC-engineered allogeneic IL-15-enhanced CD70-directed CAR-engineered NKT (^Allo^CAR70-NKT) cells

^Allo^CAR70-NKT cells were generated by differentiating gene engineered human cord blood CD34^+^ HSPCs in a 5-stage clinically guided Ex Vivo HSPC-Derived NKT Cell Culture method. The complete methodology and step-by-step protocols have been described in detail in previously published studies [17, 21]. Here, we provide a summary of the key steps involved in the culture and generation of ^Allo^CAR70-NKT cells.

At Stage 0, the frozen stock of human CD34^+^ HSPCs was thawed and cultured in T cell X-VIVO 15 Serum-Free Hematopoietic Stem Cell Medium supplemented with human Flt3L (50 ng/ml), SCF (50 ng/ml), TPO (50 ng/ml), and IL-3 (20 ng/ml) for 24 hours. Lentiviral transduction was then carried out for an additional 24 hours using the Lenti/iNKT-CAR70-IL-15 vector.

At Stage 1, gene-engineered HSPCs harvested were cultured in the feeder-free StemSpan^TM^ SFEM II Medium supplemented with StemSpan^TM^ Lymphoid Progenitor Expansion Supplement for 14 days. HSPCs were cultured in CELLSTAR^®^24-well Cell Culture Nontreated Multiwell Plates (VWR, cat. no. 82050-892). StemSpan^TM^ Lymphoid Differentiation Coating Material (500 μl/well, diluted to a final concentration of 1X from a stock dilution of 100X) was applied to the plates and left for 2 h at room temperature or overnight at 4 °C. Subsequently, 500 μl of the gene engineered CD34^+^ HSPC suspension, with a density of 2 × 10^4^ cells/ml, was added to each pre-coated well. Half of the medium in each well was removed and replaced with fresh medium twice per week.

At Stage 2, the Stage 1 cells were harvested and cultured in the feeder-free StemSpan^TM^ SFEM II Medium supplemented with StemSpan^TM^ Lymphoid Progenitor Maturation Supplement for ~7 days. StemSpan^TM^ Lymphoid Differentiation Coating Material (1 ml/well, diluted to a final concentration of 1X) was applied to Non-Treated Falcon™ Polystyrene 6-well Microplates (Thermo Fisher Scientific, cat. no. 140675); 2 ml of the harvested Stage 1 cells, resuspended with a density of 1 × 10^5^ cells/ml, was added into each pre-coated well. The cell density was maintained at 1–2  × 10^6^ cells per well during the Stage 2 culturing. Cells were passaged 2–3 times per week with the addition of fresh medium for each passage.

At Stage 3, the Stage 2 cells were harvested and cultured in the feeder-free StemSpan^TM^ SFEM II Medium supplemented with StemSpan^TM^ Lymphoid Progenitor Maturation Supplement, CD3/CD28/CD2 T Cell Activator, and human recombinant IL-15 (20 ng/ml) for 7 days. StemSpan^TM^ Lymphoid Differentiation Coating Material (1 ml/well, diluted to a final concentration of 1X) was applied to Non-Treated Falcon™ Polystyrene 6-well Microplates (Thermo Fisher Scientific, cat. no. 08-772-49); 2 ml of the harvested Stage 2 cells, resuspended with a density of 5 × 10^5^ cells/ml, was added into each pre-coated well. The cell density was maintained at 1–2 × 10^6^ cells per well during the Stage 3 culturing. Cells were passaged 2–3 times per week with the addition of fresh medium for each passage.

At Stage 4, the Stage 3 cells were harvested and verified by flow cytometry to confirm their status as mature ^Allo^CAR70-NKT cells or their derivatives; then the cells underwent expansion stage via an aAPC-based expansion. aAPC-CD70 cells were irradiated at 10,000 rads using a Rad Source RS-2000 X-Ray Irradiator (Rad Source Technologies). The Stage 3 mature ^Allo^CAR70-NKT cells and derivatives were co-cultured with the irradiated aAPCs (with a ratio of 1:1). The cells were resuspended in C10 medium supplemented with human IL- 7 (10 ng/ml) and IL-15 (10 ng/ml) at a density of 0.5–1 × 10^6^ cells/ml; 2 ml cell suspension was seeded into each well of the Corning™ Costar™ Flat Bottom Cell Culture 6-well Plates. The cell density was maintained at 0.5–1 × 10^6^ cells/ml during the expansion stage. Cells were passaged 2–3 times per week with the addition of fresh medium for each passage. The expanded ^Allo^CAR70-NKT cells were aliquoted and cryopreserved in CryoStor^®^ Cell Cryopreservation Media CS10 using a Thermo Scientific™ CryoMed™ Controlled-Rate Freezer 7450 (Thermo Scientific) for stock.

### Generation of PBMC-derived allogeneic IL-15-enhanced CD70-directed CAR-engineered NKT (^PBMC^CAR70-NKT) cells

Healthy donor PBMCs were sorted with MACS via Anti-iNKT Microbeads (Miltenyi Biotech) labeling to enrich NKT cells, following the manufacturer’s instructions. The enriched NKT cells were mixed with donor-matched irradiated αGC/PBMCs at a ratio of 1:1–1:2, followed by culturing in C10 medium supplemented with 10 ng/ml IL-7 and IL-15. On day 3, NKT cells were transduced with Lenti/CAR70-IL-15 or Lenti/CAR70-FG viruses for 24 h. The resulting ^PBMC^CAR70-NKT cells were expanded for about 2 weeks in C10 medium supplemented with 10 ng/ml IL-7 and IL-15 and cryopreserved for future use.

### Generation of PBMC-derived conventional αβ T cells

PBMCs from healthy donors were utilized to generate conventional αβ T cells, referred to as T cells. To produce T cells, PBMCs were activated using Dynabeads^TM^ Human T-Activator CD3/CD28 (Thermo Fisher Scientific, cat. no. 11131D) following the manufacturer’s guidelines. The activated cells were then cultured in C10 medium supplemented with 20 ng/ml IL-2 for a duration of 2–3 weeks.

### Generation of CD70-directed CAR-engineered conventional αβ T (CAR70-T) cells

PBMCs from healthy donors were utilized to generate CAR70-T cells. To produce these cells, non-treated tissue culture 24-well plates (Corning, cat. no. 3738) were coated with Ultra-LEAF™ Purified Anti-Human CD3 Antibody (Clone OKT3, BioLegend) at 1 µg/ml (500 µl/well), at room temperature for 2 h or at 4 °C overnight. PBMCs were resuspended in the C10 medium supplemented with 1 µg/ml Ultra-LEAF™ Purified Anti-Human CD28 Antibody (Clone CD28.2, BioLegend) and 30 ng/ml IL-2, followed by seeding in the pre-coated plates at 1 × 10^6^ cells/ml (1 ml/well). After 2 days, the cells were transduced with either Lenti/CAR70 viruses for a period of 24 h. The conventional CAR70-T cells were expanded for about 2 weeks in C10 medium and then cryopreserved for future applications.

### In vitro tumor cell killing assay

Various human AML cells (i.e., THP1-FG, KG-1-FG, and HL60-FG; 1 × 10^4^ cells per well in 96-well plate) were co-cultured with the indicated therapeutic cells (i.e., T, ^Allo^NKT, ^PBMC^NKT, ^Allo^CAR70-NKT, and ^PBMC^CAR70-NKT cells) in Corning 96-well clear bottom black plates for 24 h in C10 medium. The effector cell to target cell (E:T) ratio is indicated in the figure legends. At the end of culture, viable tumor cells were quantified by adding D-luciferin (150 μg/ml; Fisher Scientific, cat. no. 50-209-8110) to cell cultures, followed by the measurement of luciferase activity using an Infinite M1000 microplate reader (Tecan). To test NKR mediated killing, 10 μg/ ml Ultra-LEAF^TM^ purified anti-human NKG2D (Clone 1D11, BioLegend, cat. no. 320813) or anti-human DNAM-1 antibody (Clone 11A8, BioLegend, cat. no. 338302) was added to co-cultures to investigate the mechanism of tumor cell killing mediated by NKRs, and LEAF^TM^ purified mouse lgG2b κ isotype control antibody (Clone MG2b-57, BioLegend, cat. no. 401202) was included as an isotype control.

### In vitro HMA treatment assay

Human AML cell lines (i.e., THP1, KG1, and HL60) were cultured in C10 medium for 2 weeks, with or without the addition of azacitidine or decitabine. Azacitidine or decitabine was supplemented into the cell culture every two days at a concentration of 2 μM. At the end of the cell culture, AML cells were harvested and subjected to further analysis, including flow cytometry and in vitro tumor cell killing assays.

### In vivo bioluminescence live animal imaging (BLI)

BLI was conducted using the Spectral Advanced Molecular Imaging (AMI) HTX system (Spectral Instrument Imaging). Live animal images were captured 5 min after intraperitoneal (i.p.) administration of D-Luciferin, with doses of 1 mg/mouse for tumor cell visualization. The imaging data were processed and analyzed using AURA imaging software (Spectral Instrument Imaging, version 3.2.0).

### In vivo decitabine treatment study

On Day 0, NSG mice received i.v. inoculation of THP1-FG or HL60-FG human AML cells (1 × 10^6^ cells per mouse). From Day 3, mice received weekly i.p. injection of Vehicle (100 μl PBS per mouse) or decitabine (0.1 mg in 100 μl PBS per mouse). Over the experiment, mice were monitored for survival and their tumor loads were measured twice per week using BLI. At the study endpoint, mice were euthanized, and multiple tissues were harvested for flow cytometry analyses of tumor burden and antigen expression on tumor cells.

### In vivo antitumor efficacy study of ^Allo^CAR70-NKT cells: Human AML xenograft NSG mouse model

On Day 0, NSG mice received i.v. inoculation of THP1-FG, HL60-FG, or KG1-FG human AML cells (1 × 10^6^ cells per mouse). On Day 3, mice received i.p. injection of Vehicle (100 μl PBS per mouse) or decitabine (0.1 mg in 100 μl PBS per mouse). On Day 10, the experimental mice received i.v. injection of Vehicle (100 μl PBS per mouse) or ^Allo^CAR70-NKT cells (3 × 10^6^ cells in 100 μl PBS per mouse). Over the experiment, mice were monitored for survival and their tumor loads were measured twice per week using BLI. At the study endpoint, mice were euthanized, and their tumor burdens and presence of human therapeutic cells were assessed using flow cytometry. Human IFN-γ and TNF-α levels in mouse serum were measured through ELISA analyses.

### In vivo GvHD evaluation study of ^Allo^CAR70-NKT cells

On Day 0, NSG mice received i.v. injection of ^Allo^CAR70-NKT cells (10 × 10^6^ CAR^+^ cells in 100 μl PBS per mouse), ^PBMC^CAR70-NKT cells (10 × 10^6^ CAR^+^ cells in 100 μl PBS per mouse), or CAR70-T cells (10 × 10^6^ CAR^+^ cells in 100 μl PBS per mouse). Over the experiment, mice were monitored for survival, body weight, and clinical GvHD score. A score ranging from 0 to 2 was assigned for each clinical GvHD sign, which included body weight, activity, posture, skin thickening, diarrhea, and dishevelment [22]. At the end of the experiment, multiple tissues were collected and prepared for histological analysis.

### In vivo CRS evaluation study of ^Allo^CAR70-NKT cells

On Day 0, NSG mice received i.p. inoculation of THP1-FG cells (1 × 107 cells per mouse). On Day 10, the experimental mice received i.p. injection of vehicle (100 μl PBS per mouse), ^Allo^CAR70-NKT cells (10 × 106 CAR^+^ cells in 100 μl PBS per mouse), ^PBMC^CAR70-NKT cells (10 × 106 CAR^+^ cells in 100 μl PBS per mouse), or CAR70-T cells (10 × 10^6^ CAR^+^ cells in 100 μl PBS per mouse). On Day 13, blood and peritoneal fluid samples were collected from the experimental mice, and their serum and peritoneal fluid IL-6 and SAA-3 were measured using ELISA. A Mouse SAA-3 ELISA Kit (Millipore Sigma) was used to measure SAA-3, following the manufacturer’s instructions. Percentage of mouse CD11b^+^GR1^-^ macrophage in the peritoneal fluid was quantified through flow cytometry.

### In vivo dosing and toxicity evaluation study of ^Allo^CAR70-NKT cells

On Day 0, NSG mice were intravenously injected with ^Allo^CAR70-NKT cells at varying doses (1 × 10^6^, 3 × 10^6^, 5 × 10^6^, or 1 × 10^7^ CAR⁺ cells in 100 μl PBS per mouse). Body weight was monitored every 10 days throughout the study. On Day 120, major organs were collected and subjected to histopathological examination by the UCLA Pathology Core. Tissues from experimental mice that received 1 × 10^7 Allo^CAR70-NKT cells and control (blank NSG) mice were evaluated for inflammation (Inf), hematopoietic neoplasia (HN), and non-hematopoietic neoplasia (NHN). Pathological findings were scored on a 0–3 scale based on severity (0, no abnormality; 1, mild; 2, moderate; 3, severe). Data represent pathologist’s scores of individual mouse tissues.

## Results

### HMAs upregulate CD70, CD1d, and NKR ligands on AML blasts

**Fig. 1:**
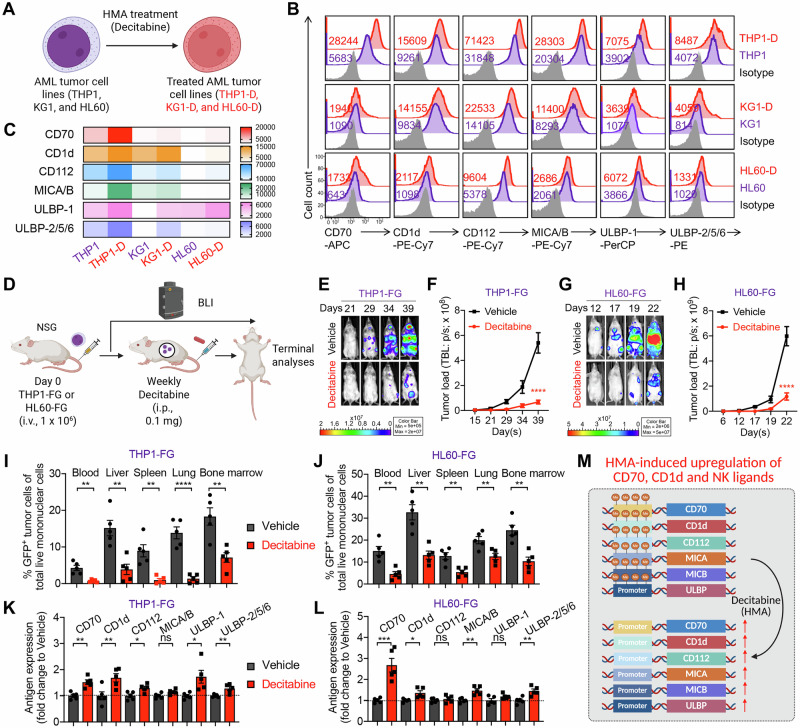
Antigen profiling of AML cells following HMA treatment in vitro and in vivo. **A**–**C** Antigen profiling of AML cells following HMA treatment in vitro. **A** Experimental design. **B** FACS detection of CAR target (CD70), NKT TCR target (CD1d), and NKR ligands (i.e., CD112, MICA/B, ULBP-1, and ULBP-2/5/6) on the indicated AML cells. **C** Heatmap showing the mean fluorescence intensity (MFI) of indicated antigen expression on AML cells. **D**–**K** Antigen profiling of AML cells following HMA treatment in vivo using THP1-FG and HL60-FG xenograft NSG mouse models. FG, firefly luciferase, and enhanced green fluorescent protein dual-reporter. **E** BLI images showing the presence of THP1-FG tumor cells in experimental mice over time. **F** Quantification of (**E**) (*n* = 5). **G** BLI images showing the presence of HL60-FG tumor cells in experimental mice over time. **H** Quantification of (**G**) (*n *= 5). **I**, **J** FACS analyses showing the percentage of tumor cells among total live mononuclear cells in the indicated tissues (*n* = 5). Mice inoculated with THP1-FG cells were euthanized on day 42, and mice inoculated with HL60-FG cells were euthanized on day 23. **K**, **L** FACS analyses showing the expression levels of the indicated antigens on tumor cells isolated from the bone marrow of experimental mice (*n* = 5). MFI values were calculated and normalized to those of tumor cells from the Vehicle group. **M** Diagram showing the upregulation of CD70, CD1d, and NK ligands on AML tumor cells following treatment with HMA. Representative of 2 (**D**–**K**) and 3 (**A**–**C**) experiments. Data are presented as the mean ± SEM. ns not significant, *p < 0.05, **p < 0.01, ***p < 0.001, ****p < 0.0001, by Student’s *t* test (**I**–**L**), or two-way ANOVA (**F** and **H**). AML acute myeloid leukemia, HMA hypomethylating agent, BLI bioluminescence imaging, i.p. intraperitoneal, FG, firefly luciferase and enhanced green fluorescent protein dual-reporter, NK natural killer.

We first confirmed that HMAs upregulate the expression of CD70, CD1d, and NKR ligands on AML cells, thereby enhancing their susceptibility to CAR70-NKT cell-mediated cytotoxicity. This was validated using both in vitro cytotoxicity assays and in vivo xenograft mouse models (Fig. 1A, D). Three representative AML cell lines—THP1, KG1, and HL60—were selected to capture the heterogeneity of antigen expression observed in AML (Fig. 1A). All three expressed high levels of NKR ligands, including MICA/B and ULBP family members (NKG2D ligands), as well as CD112 and CD155 (DNAM-1 ligands) (Fig. 1B, C). Among them, THP1 cells displayed high CD70 expression, whereas KG1 and HL60 expressed minimal CD70 (Fig. 1B, C). Both THP1 and KG1 cells showed robust CD1d expression, while HL60 cells were largely CD1d-negative (Fig. 1B, C). Together, these antigenic features highlight the diversity of AML target cell phenotypes and provide a rationale for testing the combinatorial efficacy of HMAs and CAR70-NKT cell therapy across different antigen expression contexts.

Upon long-term, low-dose HMA (i.e., decitabine) treatment in vitro, all three AML cell lines exhibited upregulation of CD70, CD1d, and NKR ligands, albeit to varying degrees but following similar overall trends (Fig. 1B, C). Notably, HL60 cells, which originally expressed minimal CD70, showed a substantial induction of CD70 expression following HMA exposure (Fig. 1B, C), suggesting that HMA treatment may sensitize otherwise CD70-low leukemic cells to CD70-targeted therapies, including monoclonal antibodies or CD70-directed CAR approaches [23, 24].

The upregulation may result from the selective survival of cells that already express higher levels of these markers, as well as intrinsic induction of these markers following decitabine treatment. To further support this, our in vitro assays show that at a later time point, when decitabine no longer affects cell viability, the AML cell lines continue to upregulate CD70, CD1d, and NKR ligands, indicating that this enhanced expression is a direct and sustained cellular response rather than an artifact of cytotoxicity (Fig. S1A–S1C).

In addition to decitabine, we further validated our conclusions using azacitidine, another HMA commonly used to treat myeloid malignancies [10, 25]. Notably, azacitidine also significantly enhanced the expression of several tumor-associated antigens, including CD70, CD1d, and multiple NKR ligands, across all three AML cell lines (Fig. S1D and S1E). However, we observed distinct expression patterns between azacitidine and decitabine, as well as variable degrees of upregulation among the three AML cell lines. These findings indicate that HMAs can differentially modulate antigen expression in a drug- and cell line–dependent manner.

Consistent with the in vitro findings, results from in vivo xenograft models using THP1 and HL60 cells further supported the modulatory effect of HMAs (Fig. 1D). Both AML cell lines were engineered to express firefly luciferase and enhanced green fluorescent protein dual-reporter (FG), allowing longitudinal monitoring of tumor burden via bioluminescence imaging (BLI). Treatment with decitabine significantly inhibited leukemia progression, as evidenced by reduced BLI signal intensity and a lower frequency of leukemic cells detected in multiple tissues (i.e., liver, lung, and bone marrow) (Fig. 1E–J). Interestingly, AML cells isolated from HMA-treated mice exhibited enhanced expression of CD70, CD1d, and several NKR ligands (Fig. 1K, L), indicating that HMA therapy reshapes the tumor antigenic landscape and may potentiate recognition and elimination by CAR70-NKT cells (Fig. 1M).

In conclusion, these findings demonstrate that HMA treatment not only directly suppresses AML progression but also enhances tumor immunogenicity by upregulating key target and co-stimulatory ligands, thereby providing a strong mechanistic rationale for combining epigenetic therapy with CAR70-NKT cell immunotherapy.

### Generate human HSPC and PBMC-derived CAR70-NKT cells

**Fig. 2:**
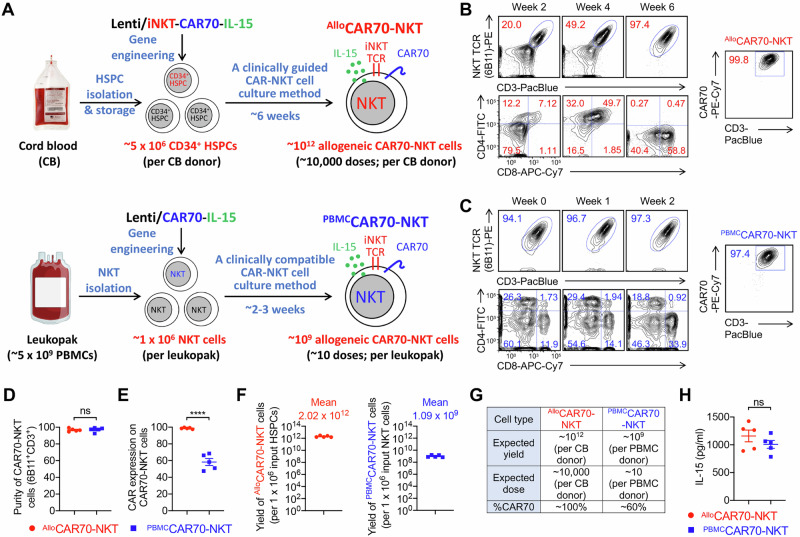
Generation of HSPC and PBMC-derived CAR70-NKT cells. **A** Diagram showing the generation of cord blood HSPC-derived allogeneic IL-15-enhanced CAR70-NKT (^Allo^CAR70-NKT) cells, as well as healthy donor PBMC-derived IL-15-enhanced CAR70-NKT (^PBMC^CAR70-NKT) cells. **B** FACS plots showing the generation of ^Allo^CAR70-NKT cells, their CD4/CD8 co-receptor profiles over the 6-week culture, and CAR70 expression on week 6 cells detected with an anti-human CD27 antibody. **C** FACS plots showing the generation of ^PBMC^CAR70-NKT cells, their CD4/CD8 co-receptor profiles over the 2-week culture, and CAR70 expression on week 2 cells detected with an anti-human CD27 antibody. Purity (**D**), CAR expression level (**E**), and yield (**F**) of ^Allo^CAR70-NKT and ^PBMC^CAR70-NKT cells (*n *= 5; n indicated different CB or PBMC donors). **G** Table showing the expected yield and dose of ^Allo^CAR70-NKT and ^PBMC^CAR70-NKT cells when delivered to cancer patients. **H** ELISA measurements of human IL-15 production by ^Allo^CAR70-NKT and ^PBMC^CAR70-NKT cells stimulated with αGC and cultured in vitro for 5 days (*n* = 5). Representative of 3 experiments. Data are presented as the mean ± SEM. ns, not significant; *p < 0.05, **p < 0.01, ****p < 0.0001, by Student’s *t* test (**D**, **E**, and **H**). HSPC hematopoietic stem and progenitor cell, NKT invariant natural killer T, CAR70 CD70-targeting chimeric antigen receptor, CB cord blood, PBMC peripheral blood mononuclear cell.

We next generated two types of CAR70-NKT cells: (1) ^Allo^CAR70-NKT cells, derived from human cord blood HSPCs using a clinically guided culture method [21]; and (2) ^PBMC^CAR70-NKT cells, derived from PBMCs obtained from healthy donors following a clinically adapted CAR-NKT manufacturing protocol (Fig. 2A) [26–28]. Because NKT cells recognize the non-polymorphic MHC class I-like molecule CD1d, they do not mediate GvHD [29–32]. Thus, both ^Allo^CAR70-NKT and ^PBMC^CAR70-NKT cells have the potential to serve as allogeneic, off-the-shelf cellular immunotherapies.

For ^Allo^CAR70-NKT cells, a lentiviral vector encoding the invariant NKT TCR (Vα24-Jα18/Vβ11), a CD70-specific CAR, and soluble IL-15 was introduced into HSPCs (Fig. 2A). The NKT TCR was cloned from healthy donor-derived NKT cells and has been previously validated to generate mature, functional NKT cells both in vitro and in vivo [19, 20, 33]. The CAR construct employs the natural CD70 ligand CD27 as its extracellular binding domain, which has been shown effective in targeting CD70-expressing malignancies such as renal cell carcinoma [18, 23, 34, 35]. Soluble IL-15 was incorporated to enhance CAR-NKT cell persistence and antitumor activity, based on extensive preclinical and clinical evidence demonstrating IL-15’s role in supporting long-term survival and function of CAR-NKT cells [36–39].

During a 6-week differentiation process, CAR-transduced HSPCs progressively generated ^Allo^CAR70-NKT cells, increasing from approximately 20% iNKT cells at week 2 to 50% at week 4, and exceeding 97% purity by week 6 (Fig. 2B). The cells underwent typical NKT developmental progression, transitioning from CD4⁻CD8⁻ double-negative (DN) to CD4⁺CD8⁺ double-positive (DP) and ultimately to DN and CD8 single-positive (SP) populations, consistent with natural NKT cell ontogeny (Fig. 2A) [40–43].

For ^PBMC^CAR70-NKT cells, PBMC-derived NKT cells were pre-sorted to achieve >95% purity, then activated and transduced with a lentiviral vector encoding the same CAR70 and IL-15 constructs (Fig. 2A, C). These cells retained their endogenous invariant TCR rather than receiving a transgenic iNKT TCR. After a 2-week expansion, the ^PBMC^CAR70-NKT products maintained a stable CD4/CD8 co-receptor distribution with CD4 SP, DN, and CD8 SP subsets, suggesting that CAR transduction and expansion did not alter lineage composition (Fig. 2C) [44–46].

Both ^Allo^CAR70-NKT and ^PBMC^CAR70-NKT products achieved >97% purity, minimizing contamination by conventional αβ T cells and thereby reducing the potential risk of GvHD (Fig. 2D). However, differences in CAR expression were noted. Because endogenous NKT cells naturally express CD27, ^PBMC^CAR70-NKT cells exhibited nearly 100% surface CD70 ligand staining, which reflects both CAR and native CD27 expression (Fig. 2C). To accurately determine the transduction rate, GFP was co-expressed from the lentivector, revealing that approximately 50–60% of ^PBMC^CAR70-NKT cells were GFP⁺, consistent with typical lentiviral transduction efficiencies observed in PBMC-derived CAR-NKT and CAR-T cell products (Fig. 2E and Fig. S2) [18]. In contrast, ^Allo^CAR70-NKT cells, which do not express endogenous CD27, showed uniform ( > 99%) CAR expression, likely due to the tricistronic lentivector design where CAR and NKT TCR are co-expressed (Fig. 2A) [18]. Positive selection during NKT differentiation favors dual CAR⁺/TCR⁺ cells, resulting in a homogeneous product with consistent CAR expression (Fig. 2B, E). These results indicate that the HSPC-based platform supports efficient and uniform CAR70-NKT cell generation.

Regarding scalability, from a single cord blood unit containing approximately 5 ×  10^6^ HSPCs, over 10^12 Allo^CAR70-NKT cells were produced, corresponding to more than 10,000 therapeutic doses based on current dosing regimens of 10^8^–10^9^ CAR-T cells per patient (Fig. 2F, G) [47, 48]. By comparison, one leukapheresis unit containing roughly 5 × 10^9^ PBMCs (including ~1 × 10^6^ NKT cells) yielded about 10^9 PBMC^CAR70-NKT cells after 2–3 weeks of culture, sufficient for treating up to 10 patients (Fig. 2F, G). These data underscore the superior scalability and manufacturing potential of the HSPC-based allogeneic CAR-NKT platform.

Furthermore, both CAR70-NKT cell types secreted high levels of IL-15 upon stimulation with the glycolipid α-galactosylceramide (αGC), confirming functional expression and cytokine secretion capability (Fig. 2H). In conclusion, both HSPC-derived and PBMC-derived CAR70-NKT cells exhibit high purity, potent expansion capacity, and robust CAR and IL-15 expression. The HSPC-engineered ^Allo^CAR70-NKT platform offers advantages in uniformity, scalability, and off-the-shelf potential, providing a strong foundation for future clinical translation in CD70⁺ malignancies.

### Characterize human HSPC and PBMC-derived CAR70-NKT cells

**Fig. 3:**
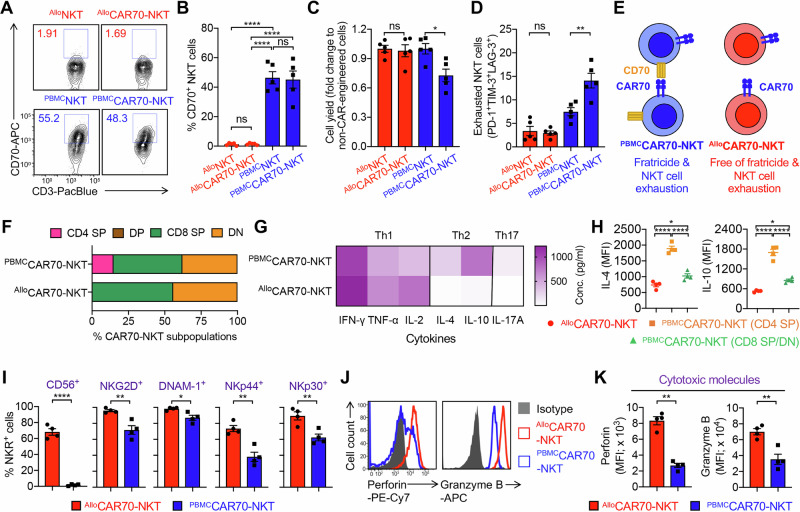
Comparison of the phenotype and functionality of HSPC and PBMC-derived CAR70-NKT cells. **A**–**E** Studying the fratricide risk of CAR70-NKT cells. **A** FACS detection of CD70 expression on ^Allo^CAR70-NKT and ^PBMC^CAR70-NKT cells. Non-CAR-engineered ^Allo^NKT and ^PBMC^NKT cells were included as controls. **B** Quantification of (**A**) (*n* = 5). **C** Fold expansion of CAR-NKT cells normalized to their non-CAR-engineered counterparts (*n* = 5). **D** Quantification of PD-1^+^LAG-3^+^TIM-3^+^ cells as a percentage of the total NKT cell population (*n* = 5). **E** Schematics showing the absence of surface CD70 expression on ^Allo^CAR70-NKT cells, indicating resistance to fratricide and reduced NKT cell exhaustion. **F** Comparison of CD4/CD8 subpopulations of the two types of CAR70-NKT cells (*n* = 5). **G** Comparison of cytokine profiles of the two types of CAR-NKT cells (*n* = 5). CAR70-NKT cells were stimulated with αGC and cultured in vitro for 5 days, and the supernatants were collected for ELISA analyses. **H** FACS detection of intracellular IL-4 and IL-10 production by the indicated CAR70-NKT cells (*n* = 4). **I** FACS analyses of surface NK markers and NKRs on the CAR70-NKT cells (*n* = 4; n indicated different CB or PBMC donors). **J** FACS plots showing the intracellular Perforin and Granzyme B production by the indicated CAR70-NKT cells. **K** Quantification of (**J**) (*n* = 4; n indicated different CB or PBMC donors). Representative of 3 experiments. Data are presented as the mean ± SEM. ns, not significant; *p < 0.05, **p < 0.01, ****p < 0.0001, by Student’s *t* test (**I** and **K**), or one-way ANOVA (**B**–**D**, and **H**). SP single-positive, DP double-positive, DN double-negative, Th1 type 1 T helper, Th2 type 2 T helper, Th17 type 17 T helper, Conc. concentration, MFI mean fluorescence intensity, NKR natural killer receptor.

We first examined CD70 expression in both ^Allo^CAR70-NKT and ^PBMC^CAR70-NKT cells, given prior findings in CD70-targeting CAR-T cell studies where endogenous CD70 expression on CAR-T cells induces fratricide and exhaustion, leading to reduced expansion and function [18, 35, 49]. Therefore, eliminating CD70 expression has been considered a key strategy to prevent self-targeting in CD70-directed CAR therapies. Interestingly, unlike ^PBMC^CAR70-NKT cells which exhibited substantial CD70 expression, ^Allo^CAR70-NKT cells did not express CD70 (Fig. 3A, B). Two additional control groups, non–CAR-engineered HSPC-derived NKT cells (^Allo^NKT) and PBMC-derived NKT cells (^PBMC^NKT), were included for comparison. Consistently, ^Allo^NKT cells lacked CD70 expression, whereas ^PBMC^NKT cells expressed it at high levels (Fig. 3A, B). These results suggest that the absence of CD70 expression is an intrinsic feature of HSPC-derived NKT cells, representing a natural advantage for CD70-targeting CAR-NKT therapy by preventing fratricide. Consistent with this, ^Allo^CAR70-NKT cells exhibited robust expansion comparable to non–CAR-engineered ^Allo^NKT cells, while ^PBMC^CAR70-NKT cells showed markedly reduced proliferation and elevated exhaustion marker expression (Fig. 3C, D). These findings indicate that the absence of CD70 expression preserves expansion potential and prevents activation-induced dysfunction in ^Allo^CAR70-NKT cells (Fig. 3E).

We next analyzed the cytokine profiles of CAR70-NKT cells. ^Allo^CAR70-NKT cells contained predominantly DN and CD8 SP subsets, lacking the CD4 SP population commonly observed in ^PBMC^CAR70-NKT cells (Fig. 3F). Since CD4 SP NKT cells are generally associated with helper and regulatory functions and are major producers of Th2/Th17 cytokines, their absence in ^Allo^CAR70-NKT cells may contribute to a more cytotoxic and Th1-skewed phenotype [50–52]. Indeed, cytokine profiling revealed that AlloCAR70-NKT cells produced higher or comparable levels of Th1 cytokines (IFN-γ, TNF-α, and IL-2) but significantly lower levels of Th2/Th17 cytokines (IL-4, IL-10, and IL-17A), which were predominantly generated by CD4 SP ^PBMC^CAR70-NKT cells (Fig. 3G, H). This Th1-dominant cytokine pattern aligns with a proinflammatory, antitumor phenotype favorable for tumor targeting [53–56].

Furthermore, flow cytometry analyses demonstrated that ^Allo^CAR70-NKT cells expressed higher levels of NK-associated markers and activating receptors, including CD56, NKG2D, DNAM-1, NKp44, and NKp30, compared with ^PBMC^CAR70-NKT cells (Fig. 3I). Correspondingly, ^Allo^CAR70-NKT cells exhibited enhanced cytotoxic potential, evidenced by elevated expression of Perforin and Granzyme B (Fig. 3J, K). Collectively, these results indicate that ^Allo^CAR70-NKT cells possess a less exhausted, Th1-skewed, and highly cytotoxic phenotype, making them a promising off-the-shelf cellular therapy platform for targeting CD70⁺ malignancies without the risk of fratricide.

### ^Allo^CAR70-NKT cells exhibit potent antitumor activity against AML in vitro and synergize with HMAs

We then evaluated the antitumor activity and potential synergistic mechanisms of CAR70-NKT cells in vitro using a panel of AML cell lines (THP1-FG, HL60-FG, and KG1-FG) as target cells (Fig. 3A). These AML lines represent distinct antigen-expression profiles, with or without treatment with decitabine, which upregulates CD70, CD1d, and NK ligands (Fig. 3B). Five types of effector cells were included: ^Allo^CAR70-NKT, ^PBMC^CAR70-NKT, non-CAR-engineered ^Allo^NKT, non-CAR-engineered ^PBMC^NKT, and conventional PBMC-derived T cells. Additionally, αGC was included in selected co-cultures to stimulate NKT TCR/CD1d-mediated cytotoxicity.

As expected, conventional T cells exhibited minimal tumor killing under all conditions due to the absence of CAR, TCR-mediated CD1d recognition, or NKR-mediated cytotoxicity (Fig. S3A). ^PBMC^NKT cells did not kill AML targets without αGC, but αGC stimulation enabled killing of CD1d^+^ tumor cells, indicating that ^PBMC^NKT-mediated cytotoxicity relies primarily on TCR/CD1d recognition and lacks strong NKR-mediated killing (Fig. S3A). In contrast, ^Allo^NKT cells efficiently killed AML cells in the absence of both αGC and CD1d expression, demonstrating robust NKR-mediated tumor recognition and cytotoxicity. This mechanism was consistent with their high expression of NKRs and cytotoxic molecules and was confirmed in NKR-blocking experiments, where simultaneous blockade of NKG2D and DNAM-1 significantly reduced their antitumor activity (Fig. 3I–K and Fig. S3B).

Both ^Allo^CAR70-NKT and ^PBMC^CAR70-NKT cells efficiently killed CD70^+^ AML cells and CD1d^+^ targets in the presence of αGC (Fig. S4A). Notably, only ^Allo^CAR70-NKT cells were capable of killing CD70^–^CD1d^–^ AML cells via NKR-mediated recognition, highlighting a triple-targeting mechanism through CAR, TCR/CD1d, and NKRs (Fig. S3A and S3C). ^PBMC^CAR70-NKT cells, in contrast, lacked NKR-mediated killing (Fig. S3A and S3C).

**Fig. 4:**
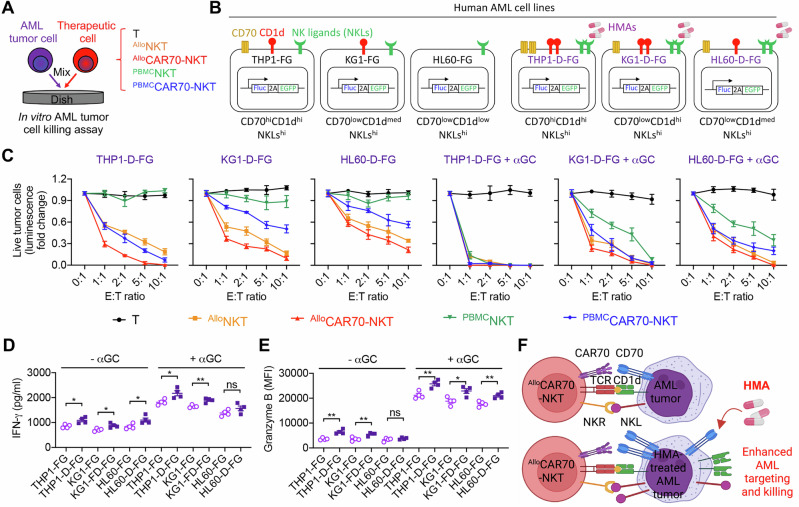
Synergistic effect of CAR70-NKT cells with HMA in the treatment of AML in vitro. **A** Experimental design to study the synergistic effect of CAR70-NKT cells with Decitabine. **B** Schematics showing the indicated human AML cell lines. **C** Tumor cell killing data at 24 h (*n* = 4). AML cells were pretreated with decitabine and thoroughly washed prior to the cytotoxicity assay to ensure removal of residual drug, as decitabine is cytotoxic to therapeutic cells. **D** ELISA analysis of IFN-γ production by ^Allo^CAR70-NKT cells 24 h after co-culture with the indicated AML tumor cells, in the presence or absence of αGC (*n* = 4). **E** FACS analyses of intracellular production of Granzyme B by ^Allo^CAR70-NKT cells 24 h after co-culture with the indicated AML tumor cells, in the presence or absence of αGC (*n* = 4). **F** Diagram illustrating the upregulation of CD70, CD1d, and NK ligands on AML tumor cells treated with HMA, leading to enhanced tumor recognition and killing by ^Allo^CAR70-NKT cells. Representative of 3 experiments. Data are presented as the mean ± SEM. ns, not significant, *p < 0.05, **p < 0.01, ***p < 0.001, ****p < 0.0001, by Student’s *t* test (**D** and **E**). NKL natural killer ligand, E:T effector-to-tumor cell ratio, TCR T cell receptor, αGC α-Galactosylceramide.

Treatment of AML cells with decitabine enhanced tumor killing by all effector cell types, reflecting increased target antigen expression (Fig. 4C). For example, ^PBMC^CAR70-NKT cells, which were initially unable to kill HL60-FG cells without αGC, demonstrated potent cytotoxicity following decitabine-induced upregulation of CD70, CD1d, and NK ligands (Fig. 4C). Of note, ^Allo^CAR70-NKT cells exhibited elevated IFN-γ production and Granzyme B expression when targeting decitabine-treated AML cells (Fig. 4D, E).

These results highlight a synergistic effect between CAR70-NKT cells and HMA treatment. ^Allo^CAR70-NKT cells utilize CAR, TCR/CD1d, and NKR pathways for broad AML killing. Decitabine upregulated CD70, CD1d, and NK ligands, enhancing cytotoxicity of both ^Allo^CAR70-NKT and ^PBMC^CAR70-NKT cells (Fig. 4F). This combination achieves enhanced, multi-modal tumor cell killing, overcoming antigen heterogeneity and immune evasion in AML.

Lastly, we performed a side-by-side comparison of ^Allo^CAR70-NKT cell-mediated tumor killing against AML cells pretreated with either decitabine or fludarabine. Fludarabine, a commonly used chemotherapeutic agent in AML, exerts primarily cytotoxic rather than epigenetic effects and therefore is not expected to induce substantial changes in antigen expression [57]. Consistent with this, ^Allo^CAR70-NKT cells exhibited markedly enhanced cytotoxicity toward decitabine-treated AML cells compared with untreated controls, whereas no improvement in killing efficiency was observed following fludarabine pretreatment (Fig. S4A and S4B). These results indicate that antigen upregulation induced by HMAs, rather than general cytotoxic stress, is a key contributor to the enhanced susceptibility of AML cells to ^Allo^CAR70-NKT cell mediated killing.

### ^Allo^CAR70-NKT cells display robust in vivo antileukemic efficacy across multiple AML models and synergize with HMAs

**Fig. 5:**
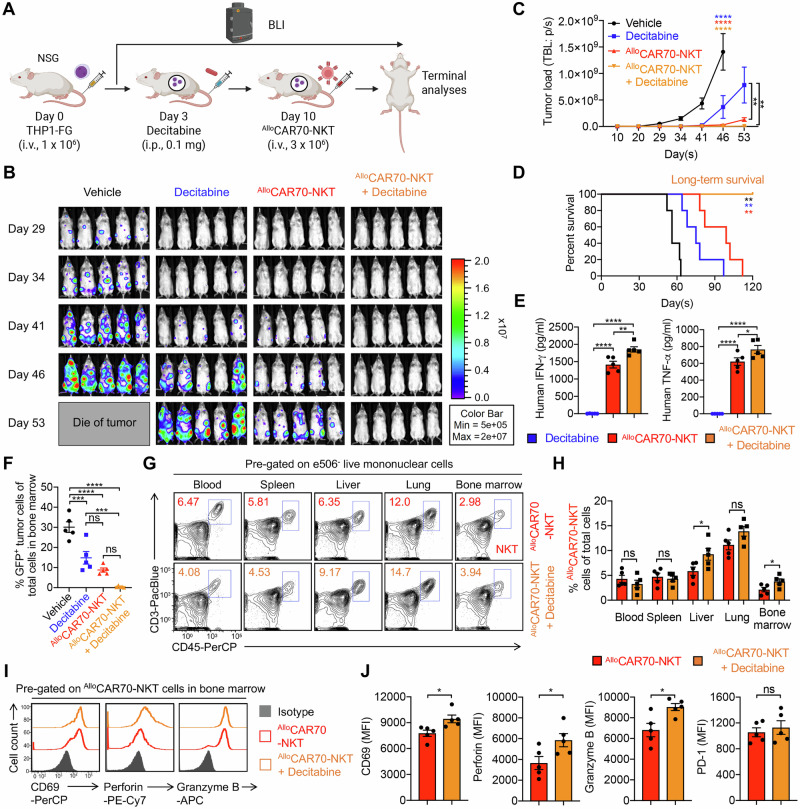
Synergistic effect of ^Allo^CAR70-NKT cells with HMA in the treatment of CD70-positive AML using a THP1-FG xenograft mouse model. **A** Experimental design to study the synergistic effect of ^Allo^CAR70-NKT cells with Decitabine in vivo. A human CD70^+^ AML cell line THP1-FG was utilized. **B** BLI images showing the presence of THP1-FG tumor cells in experimental mice over time. **C** Quantification of (**B**) (*n* = 5). **D** Kaplan–Meier survival curves (*n* = 5). **E** ELISA analyses of human IFN-γ and TNF-α levels in serum collected from experimental mice on day 20. **F** FACS analyses of the percentage of tumor cells (identified as GFP^+^ cells) among total live cells in bone marrow collected from experimental mice at the study endpoint. **G** FACS detection of ^Allo^CAR70-NKT cells in mouse tissues on day 35. **H** Quantification of (**G**) (*n* = 5). **I** FACS detection of CD69 expression, as well as Perforin and Granzyme B production by ^Allo^CAR70-NKT cells in mouse bone marrow on day 35. **J** Quantification of (**I**) (*n* = 5). Representative of 3 experiments. Data are presented as the mean ± SEM. ns, not significant, *p < 0.05, **p < 0.01, ***p < 0.001, ****p < 0.0001, by Student’s *t* test (**H** and **J**), one-way ANOVA (**E** and **F**), two-way ANOVA (**C**), or log rank (Mantel-Cox) test adjusted for multiple comparisons (**D**). i.v. intravenous, TBL total bioluminescence.

**Fig. 6:**
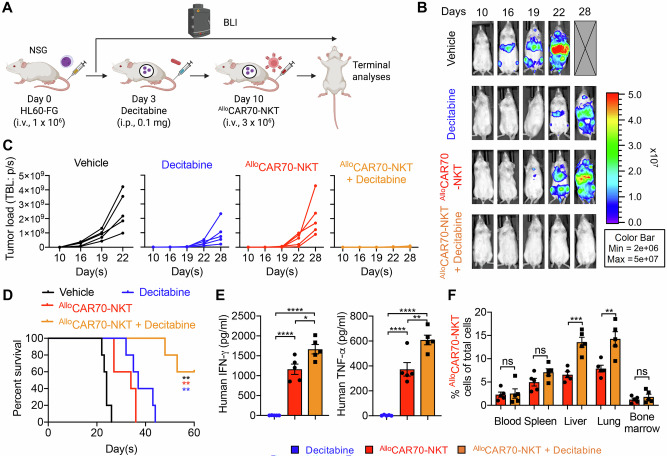
Synergistic effect of ^Allo^CAR70-NKT cells with HMA in the treatment of CD70-negative AML using a HL60-FG xenograft mouse model. **A** Experimental design to study the synergistic effect of ^Allo^CAR70-NKT cells with Decitabine in vivo. A human CD70^-^ AML cell line HL60-FG was utilized. **B** BLI images showing the presence of HL60-FG tumor cells in experimental mice over time. **C** Quantification of (**B**) (*n* = 5). **D** Kaplan–Meier survival curves (*n* = 5). **E** ELISA analyses of human IFN-γ and TNF-α levels in serum collected from experimental mice on day 18. **F** FACS analyses of the percentage of ^Allo^CAR70-NKT cells among total live mononuclear cells in mouse tissues on day 25 (*n* = 5). Representative of 3 experiments. Data are presented as the mean ± SEM. ns not significant, *p < 0.05, **p < 0.01, ***p < 0.001, ****p < 0.0001, by Student’s *t* test (**F**), one-way ANOVA (**E**), or log rank (Mantel-Cox) test adjusted for multiple comparisons (**D**).

We evaluated the antitumor efficacy of ^Allo^CAR70-NKT cells in vivo using two AML xenograft models: THP1-FG, representing CD70^high^ tumor cells (Fig. 5), and HL60-FG, representing CD70^low^ tumor cells (Fig. 6). In both models, AML cells were intravenously injected, leading to systemic dissemination to multiple organs, including liver, lung, and bone marrow, ultimately resulting in mortality. Decitabine was administered on Day 3, followed by ^Allo^CAR70-NKT cell infusion on Day 10 to target residual or relapsed AML cells (Figs. 5A and 6A).

In the THP1-FG xenograft model, decitabine treatment partially controlled tumor growth and extended survival; however, residual tumor cells persisted and upregulated CD70, CD1d, and NK ligands (Fig. 1E–K, and 5B–D). Subsequent ^Allo^CAR70-NKT cell therapy achieved complete tumor elimination in all animals, resulting in long-term survival (Fig. 5B–D). This response was associated with robust in vivo effector cytokine production (IFN-γ and TNF-α) and the absence of detectable tumor cells in key tissues such as the bone marrow (Fig. 5E, F).

Analysis of ^Allo^CAR70-NKT cell biodistribution and persistence revealed comparable frequencies in blood, spleen, and lung, with significantly higher accumulation in liver and bone marrow (Fig. 5G, H). These cells exhibited elevated activation markers (CD69) and cytotoxic molecules (Perforin and Granzyme B) while maintaining similar levels of the exhaustion marker PD-1, indicating potent and sustained effector function in target tissues (Fig. 5I, J).

Notably, ^Allo^CAR70-NKT cells exhibited markedly superior tumor control compared with unmodified ^Allo^NKT cells, as reflected by substantially reduced leukemic burden and prolonged survival in treated animals (Fig. S5A–S5C). These findings underscore the importance of CAR70 engineering in enhancing the antitumor potency of NKT cells, enabling more efficient recognition and elimination of CD70⁺ AML cells in vivo.

In the HL60-FG xenograft model, ^Allo^CAR70-NKT cells demonstrated limited efficacy against CD70^low^ tumor cells when administered alone (Fig. 6B–D). However, combination with decitabine significantly enhanced tumor control, prolonged survival, increased effector cytokine production, and promoted expansion of ^Allo^CAR70-NKT cells in organs such as liver and lung (Fig. 6B–F). To further substantiate the requirement for HMA priming in CD70^low^ AML, we expanded our analyses to include an additional CD70^low^ AML cell line, KG1-FG, which exhibited similarly low basal CD70 expression as HL60-FG cells (Fig. 1B, C). Consistently, decitabine treatment induced enhanced KG1-FG susceptibility to ^Allo^CAR70-NKT cell-mediated killing (Fig. S6A–S6D). Together, these results demonstrate that HMA priming is required in CD70^low^ AML contexts to enable efficient ^Allo^CAR70-NKT cell targeting and anti-leukemic activity.

Overall, these findings demonstrate that ^Allo^CAR70-NKT cells mediate robust antitumor activity in vivo, and that synergistic treatment with HMAs enhances tumor recognition, effector function, and survival, even against antigen-low or CD70-negative AML cells.

### ^Allo^CAR70-NKT cells demonstrate a favorable safety profile in vivo

Lastly, we evaluated the safety profile of CAR70-NKT cells, focusing on GvHD, CRS, and long-term tissue toxicity, as these are major concerns for the translational and clinical development of allogeneic cell therapies [58–61]. Healthy donor PBMC-derived conventional CAR70-engineered T (CAR70-T) cells were included as a positive control (Fig. S7).

**Fig. 7:**
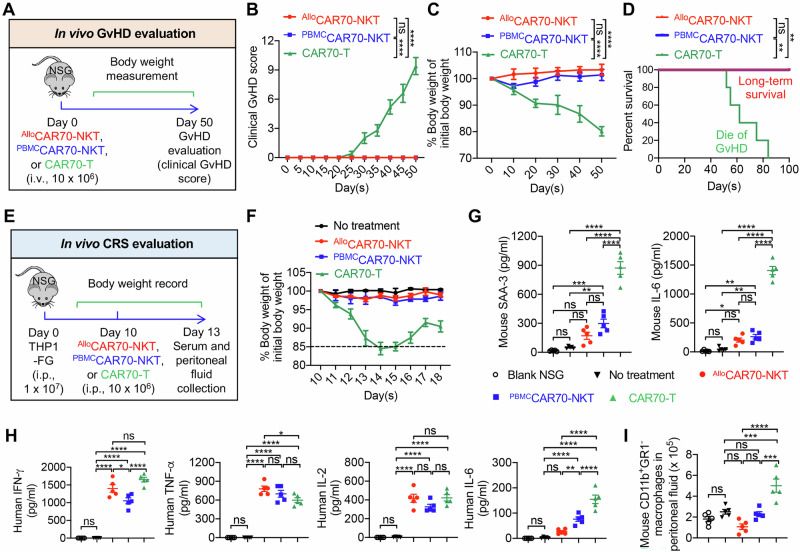
Safety evaluation of CAR70-NKT cells. **A**–**D** Studying the GvHD risk of CAR70-NKT cells using a human xenograft NSG mouse model. Conventional CD70-targeting CAR-engineered T (CAR70-T) cells were included as a control. **A** Experimental design. **B** Clinical GvHD score recorded over time (*n *= 5). The score was calculated as the sum of individual scores of 6 categories (body weight, activity, posture, skin thickening, diarrhea, and dishevelment; score 0–2 for each category). **C** Body weight measured over time (*n* = 5). **D** Kaplan–Meier survival curves (*n* = 5). **E**–**I** Studying the CRS toxicity induced by CAR70-NKT cells using a THP1-FG human AML xenograft NSG mouse model. **E** Experimental design. **F** Body weight of experimental mice over time (*n* = 5). **G** ELISA analyses of mouse IL-6 and SAA-3 in mouse serum (*n* = 5). **H** ELISA analyses of human cytokines (i.e., IFN-γ, TNF-α, IL-2, and IL-6) in mouse serum (*n *= 5). **I** Quantification of mouse peritoneal macrophage numbers from the indicated samples. Mouse macrophages were identified as mouse CD45^+^CD11b^+^GR1^-^ cells. Representative of 3 experiments. Data are presented as the mean ± SEM. ns, not significant, *p < 0.05, **p < 0.01, ***p < 0.001, ****p < 0.0001, by one-way ANOVA (**G**–**I**), two-way ANOVA (**B** and **C**), or log rank (Mantel-Cox) test adjusted for multiple comparisons (**D**). GvHD graft-versus-host disease, CRS cytokine release syndrome, SAA-3 serum amyloid A 3.

GvHD is a primary safety concern for allogeneic cell therapies [62–64]. We assessed xenogeneic GvHD in NSG mice by intravenously injecting three types of therapeutic cells and monitoring the development of GvHD (Fig. 7A). Conventional CAR70-T cells rapidly induced significant xenogeneic GvHD, as evidenced by weight loss, elevated clinical GvHD scores, and early mortality (Fig. 7B–D). In contrast, both ^Allo^CAR70-NKT and ^PBMC^CAR70-NKT cells did not induce GvHD (Fig. 7B–D). This is consistent with previous preclinical studies and clinical reports, due to their TCRs recognizing CD1d rather than polymorphic MHC, limiting alloreactivity [22, 27, 29, 30, 65, 66].

CRS is a common toxicity associated with CAR-T cell therapy, triggered by overactivation of immune effector cells and excessive proinflammatory cytokine release [67–69]. Using a high-tumor-load xenograft mouse model where tumor cells and therapeutic cells were co-injected intraperitoneally (Fig. 7E) [67], conventional CAR70-T cells caused severe CRS, evidenced by rapid body weight loss and elevated serum levels of CRS-associated biomarkers, including mouse SAA-3 and IL-6 (Fig. 7F, G). In contrast, both ^Allo^CAR70-NKT and ^PBMC^CAR70-NKT cells did not induce these responses, indicating a lower CRS risk (Fig. 7F, G). Notably, ^Allo^CAR70-NKT cells produced comparable or higher levels of human Th1 cytokines (IFN-γ, TNF-α, IL-2) relative to ^PBMC^CAR70-NKT and CAR70-T cells, but did not generate human IL-6, unlike the other cell types (Fig. 7H). The low CRS potential of CAR70-NKT cells likely reflects their NK-like features and their capacity to deplete macrophages in vivo that drive CRS (Fig. 7I) [37, 38, 55, 70].

To assess potential dose-dependent toxicity, ^Allo^CAR70-NKT cells were administered intravenously at 1, 3, 5, or 10 × 10^6^ cells per mouse (Fig. S8A). No acute toxicity was observed (Fig. S8B and S8C). Long-term tissue analyses conducted 120 days post-injection revealed no detectable organ toxicity or histopathological abnormalities (Fig. S8D). Overall, these studies demonstrate that ^Allo^CAR70-NKT cells exhibit a high safety profile, with no detectable GvHD, minimal CRS risk, and no long-term tissue toxicity, supporting their translational and clinical development as a potent and safer alternative to conventional CAR-T cell therapy in the treatment of cancers including AML.

## Discussion

In this study, we aimed to develop an allogeneic CD70-targeting CAR-NKT cell therapy for the treatment of AML, in combination with HMAs such as decitabine. Analysis of AML cells following HMA treatment revealed upregulation of key antigens, including CD70, CD1d, and NK receptor ligands, providing a strong scientific rationale for combining HMAs with CAR-NKT cell therapy to enhance tumor recognition and killing (Fig. 1). We performed a side-by-side comparison of two CAR70-NKT cell products: HSPC-derived allogeneic CAR70-NKT cells, generated using a clinically guided culture method and currently under preclinical development [17, 18, 71], and PBMC-derived CAR70-NKT cells, produced using a clinical-compatible culture protocol [17, 27, 36]. We evaluated their manufacturing feasibility, in vitro and in vivo antitumor activity, safety profile, and synergistic potential with HMAs. Overall, ^Allo^CAR70-NKT cells demonstrated superior yield, purity, antitumor efficacy, multi-modal tumor-targeting capacity, and safety, supporting their further development as a promising off-the-shelf immunotherapy for AML.

Although HMAs have become a cornerstone of standard clinical therapy for AML, prolonged treatment is frequently associated with the emergence of drug resistance [7]. Resistance to HMAs can be broadly classified into two categories: primary resistance, in which patients fail to respond after at least 4–6 treatment cycles, and secondary (or acquired) resistance, which occurs when initially responsive patients relapse following extended therapy. Mechanistically, resistance is driven by factors such as alterations in drug metabolism, epigenetic plasticity, activation of compensatory signaling pathways, and changes in the bone marrow microenvironment [7, 25, 72, 73]. To overcome these limitations, recent studies have investigated combination strategies that pair HMAs with targeted therapies, immunotherapies, or additional epigenetic modulators, with the goal of enhancing response rates and prolonging survival in AML patients who are ineligible for intensive chemotherapy. Continued investigation is critical to refine HMA-based regimens and address the challenges posed by disease heterogeneity and therapeutic resistance.

^Allo^CAR70-NKT cell therapy represents a novel and promising approach to synergize with HMAs in the treatment of AML. First, HMAs can induce upregulation of tumor-associated antigens, including CD70, on AML cells, which enhances the recognition and cytotoxic activity of ^Allo^CAR70-NKT cells (Fig. 1). This antigen modulation not only improves immediate tumor killing but also promotes in vivo persistence and sustained functionality of the infused cells, potentially extending the duration of therapeutic benefit (Figs. 5 and 6). Second, ^Allo^CAR70-NKT cells possess multiple tumor-targeting mechanisms beyond CD70 recognition, enabling them to eliminate AML cells with low or absent CD70 expression (Figs. 4, 6). This multi-pronged targeting reduces the likelihood of tumor escape and disease relapse due to antigen loss, a common limitation in single-target CAR therapies [74–76]. Third, preclinical studies indicate that ^Allo^CAR70-NKT cells exhibit a favorable safety profile, with minimal risk of GvHD or other systemic toxicities, making them a safer alternative to conventional allogeneic T cell therapies (Fig. 7). Finally, ^Allo^CAR70-NKT cells can be manufactured at high yield and purity, allowing for an off-the-shelf, ready-to-use product. Their scalability and cost-effectiveness make them particularly well-suited for AML patients who are ineligible for intensive chemotherapy or who require rapid intervention [59, 77–79]. Taken together, the combination of HMAs with ^Allo^CAR70-NKT cells offers a compelling strategy to enhance anti-leukemic efficacy while addressing limitations of current therapies, including resistance, antigen escape, and treatment-related toxicity.

Although promising, several future directions could be pursued to further enhance the therapeutic potential of ^Allo^CAR70-NKT cell therapy for AML and to optimize its synergy with HMAs. In the current study, IL-15 was incorporated into the CAR construct to promote CAR-NKT cell persistence and in vivo functionality. Interestingly, while IL-15–engineered conventional CAR-T cells have been associated with CRS and systemic toxicity—as observed in a clinical trial evaluating GPC3-targeting CAR-T cells for hepatocellular carcinoma—IL-15 appears to be well-tolerated in the context of CAR-NKT cell therapy [27, 39, 80]. Beyond IL-15, other cytokines such as IL-12, IL-18, and IL-21 have been investigated to enhance CAR-based immunotherapies through mechanisms including improved effector function, resistance to exhaustion, and modulation of the tumor microenvironment [81–84]. Notably, a recent clinical trial in lymphoma demonstrated that patients receiving a second infusion of IL-18-engineered CD19-targeting CAR-T cells, following relapse or resistance to prior CAR-T therapy, exhibited restored CAR-T functionality and potent antitumor responses without significant toxicity [85]. These findings highlight the potential of IL-18 as a powerful immunomodulatory cytokine to overcome CAR-cell dysfunction [86]. Therefore, engineering IL-18-expressing ^Allo^CAR70-NKT cells represents a compelling next step that warrants further investigation to determine whether this approach could augment antileukemic efficacy while maintaining a favorable safety profile.

In addition, as an allogeneic cell therapy, immunogenicity and host-mediated allorejection must be carefully considered [64, 87–89]. HSPC-derived ^Allo^CAR-NKT cells naturally exhibit low expression of HLA class I, HLA class II, and NK cell–activating ligands, conferring a hypoimmunogenic phenotype that renders them relatively resistant to rejection by host T and NK cells [17, 30, 90]. If necessary, further genetic modifications can be introduced to enhance immune evasion, such as targeted deletion of HLA class I and II molecules to prevent T cell–mediated rejection, combined with enforced expression of non-polymorphic HLA-E to protect against NK cell–mediated cytotoxicity [30]. Overall, the flexibility of the HSPC engineering platform enables multiplex genetic modifications, providing a robust strategy to optimize both the persistence and safety of ^Allo^CAR70-NKT cells for universal application in AML and other malignancies.

Another limitation of this study is the lack of experiments using primary AML samples or patient-derived xenograft (PDX) models. While AML cell line models provide valuable mechanistic insight and allow controlled comparisons, they do not fully recapitulate the biological diversity, genetic heterogeneity, or microenvironmental complexity seen in patient tumors. Primary AML samples would enable evaluation of donor-to-donor variability and antigen expression patterns, whereas PDX models would offer a more physiologically relevant in vivo platform to assess efficacy, persistence, and therapeutic resistance. Incorporating these systems in future studies will be essential to further strengthen and validate the translational relevance of ^Allo^CAR70-NKT cell therapy.

## Supplementary information

Supplementary figures

Supplementary methods

## Data Availability

The data generated and analyzed during this study are included in the article. Additional data generated during the current study are available from the corresponding author upon reasonable request.
