# Supplementary material for: Synergizing hypomethylating agents with off-the-shelf CD70-targeted chimeric antigen receptor-engineered natural killer T cells for the treatment of acute myeloid leukemia
Source: Leukemia. 2026 Mar 26;40(5):880–93. doi: 10.1038/s41375-026-02930-5 (PMC13149322; doi:10.1038/s41375-026-02930-5)
Supplement: Supplementary file 1 — Supplementary figures [file 41375_2026_2930_MOESM1_ESM.pdf]

Supplementary figures

Synergizing hypomethylating agents with off-the-shelf CD70-targeted chimeric antigen receptor-engineered natural killer T cells for the treatment of acute myeloid leukemia

Yan-Ruide Li[1,2], Xinyuan Shen[1,2], Yuning Chen[1,2], Yichen Zhu[1,2], Jie Huang[1,2], Caspian Oliai[3], Lili Yang[1,2,4,5,6,7,8,§]

Author Affiliation:

[1]Department of Microbiology, Immunology & Molecular Genetics, University of California, Los Angeles, Los Angeles, CA 90095, USA

[2]Department of Bioengineering, University of California, Los Angeles, Los Angeles, CA 90095, USA

[3]Division of Hematology-Oncology, Department of Medicine, David Geffen School of Medicine, University of California, Los Angeles, Los Angeles, CA 90095, USA

[4]Jonsson Comprehensive Cancer Center, David Geffen School of Medicine, University of California, Los Angeles, Los Angeles, CA 90095, USA

[5]Eli and Edythe Broad Center of Regenerative Medicine and Stem Cell Research, University of California, Los Angeles, Los Angeles, CA 90095, USA

[6]Molecular Biology Institute, University of California, Los Angeles, CA 90095, USA

[7]Parker Institute for Cancer Immunotherapy, University of California, Los Angeles, Los Angeles, CA 90095, USA

[8]Goodman-Luskin Microbiome Center, University of California, Los Angeles, Los Angeles, CA 90095, USA

[§]Corresponding author. Email: liliyang@ucla.edu

Address Correspondence to:

Lili Yang, Ph.D.

Department of Microbiology, Immunology & Molecular Genetics

University of California, Los Angeles

Los Angeles, CA 90095, USA.

Phone: 310-825-8609, Email: liliyang@ucla.edu

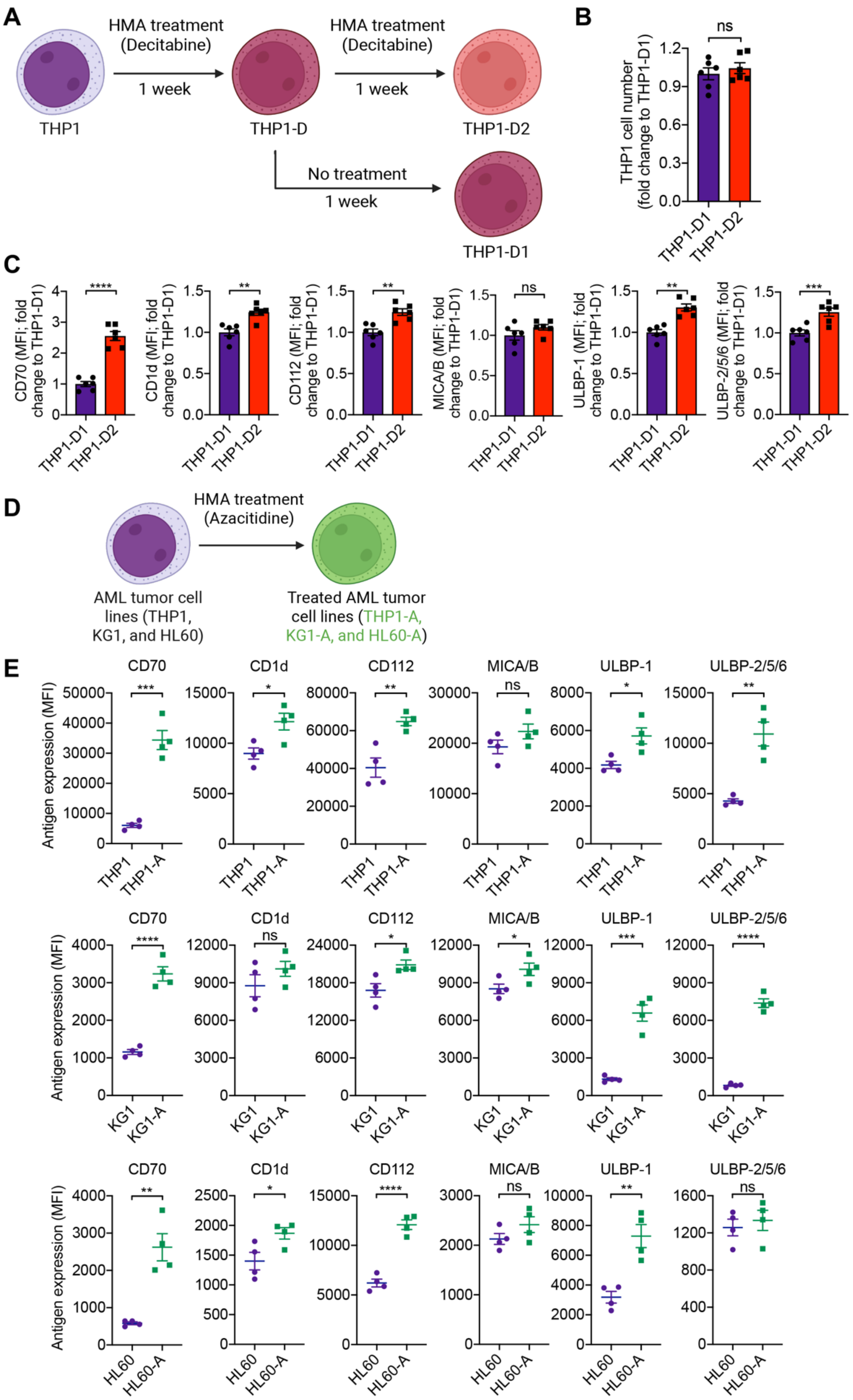

Figure S1. Antigen profiling of AML tumor cells following HMA treatment *in vitro*.

(A-C) Antigen profiling of THP1 AML cells following decitabine treatment *in vitro*. (A) Experimental design. (B) Quantification of THP-1 cell numbers. Values were normalized to the

corresponding THP1-D1 cell numbers (n = 6). (C) FACS analyses of the expression of CAR target (CD70), NKT TCR target (CD1d), and NKR ligands (i.e., CD112, MICA/B, ULBP-1, and ULBP-2/5/6) on the indicated AML cells. Values were normalized to the corresponding THP1-D1 cells (n = 6).

(D and E) Antigen profiling of AML tumor cells following azacitidine treatment *in vitro*. (D) Experimental design. (E) FACS analyses of CAR target (CD70), NKT TCR target (CD1d), and NKR ligands (i.e., CD112, MICA/B, ULBP-1, and ULBP-2/5/6) on the indicated AML cells (n = 4).

Representative of 3 experiments. Data are presented as the mean ± SEM. ns, not significant, *p < 0.05, **p < 0.01, ***p < 0.001, ****p < 0.0001, by Student's *t* test.

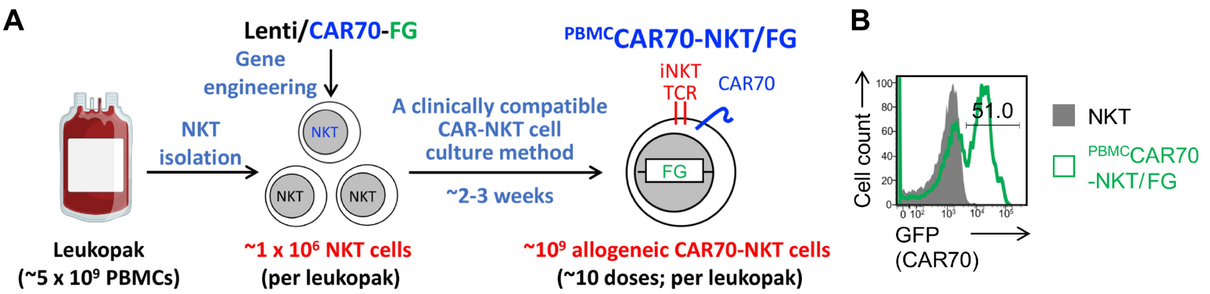

Figure S2. Generation of FG-labled PBMC-derived CAR70-NKT (^{PBMC}CAR70-NKT/FG) cells.

(A) Diagram showing the generation of ^{PBMC}CAR70-NKT/FG cells.

(B) FACS plots showing the CAR70 expression on ^{PBMC}CAR70-NKT/FG cells. Representative of 5 experiments.

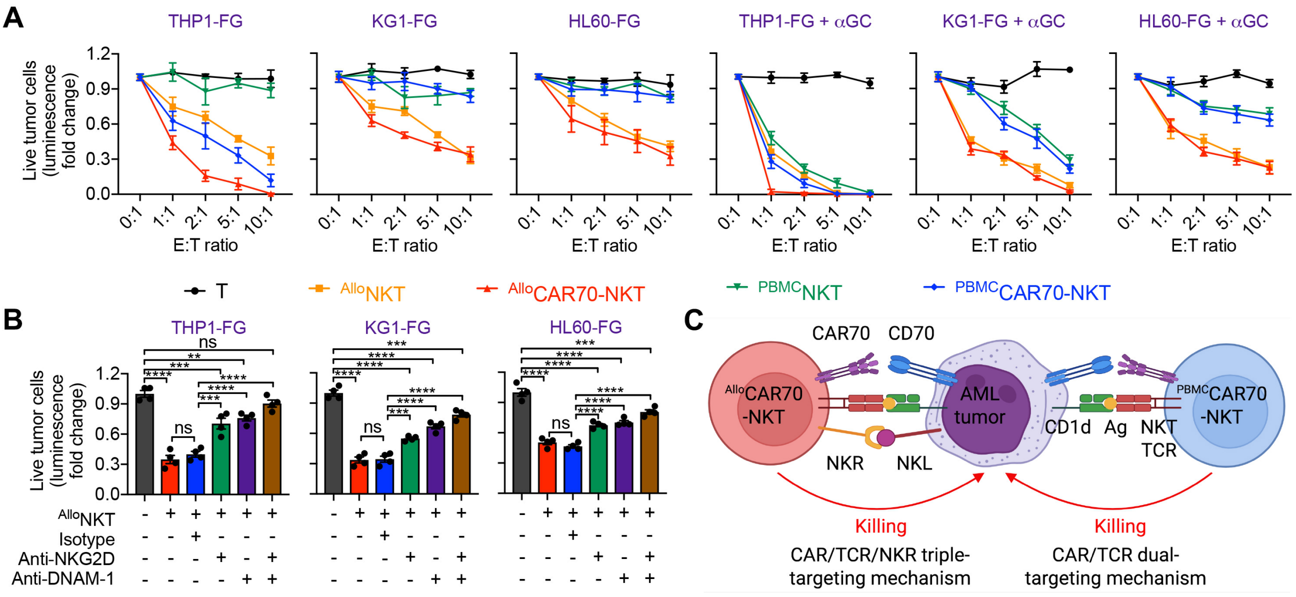

Figure S3. Studying the *in vitro* antitumor capacity of CAR70-NKT cells.

(A) Tumor cell killing data at 24 h (n = 4).

(B) Studying the tumor cell killing mechanisms of ^{Allo}NKT cells mediated by NKRs (i.e., NKG2D and DNAM-1). Tumor cell killing data at 24 h (E:T ratio = 10:1; n = 4).

(C) Diagram showing the CAR/TCR/NKR triple tumor-targeting mechanisms of ^{Allo}CAR70-NKT cells, and the CAR/TCR dual tumor-targeting mechanism of ^{PBMC}CAR70-NKT cells.

Representative of 3 experiments. Data are presented as the mean ± SEM. ns, not significant, **p < 0.01, ***p < 0.001, ****p < 0.0001, by one-way ANOVA (B).

Abbreviations: Ag antigen.

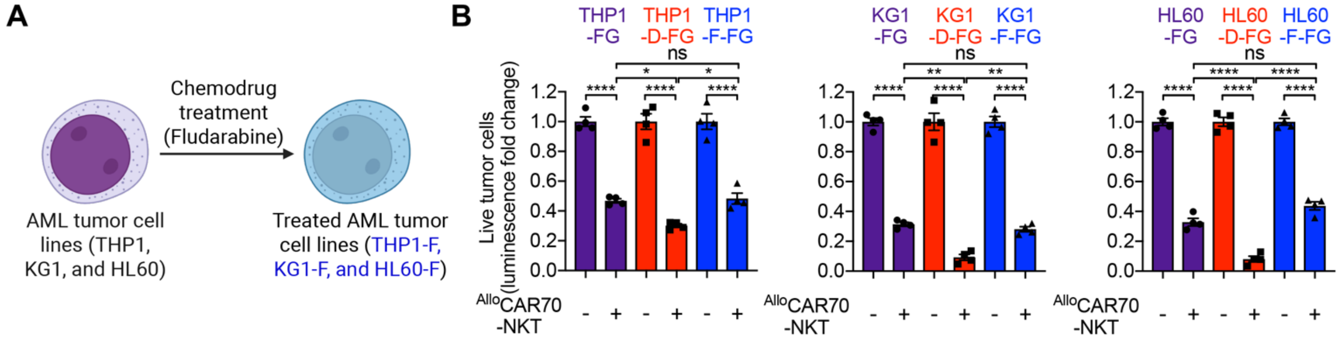

Figure S4. Comparison of ^{Allo}CAR70-NKT cell-mediated tumor killing against AML cells pretreated with either decitabine or fludarabine.

(A) Diagram showing the generation of fludarabine-treated AML tumor cells.

(B) Tumor cell killing data at 24 h (n = 4). THP1, E:T = 1:1; KG1, E:T = 10:1; HL60, E:T = 10:1, and αGC was added to the HL60 tumor cell killing coculture.

FG denotes a dual reporter consisting of firefly luciferase and enhanced green fluorescent protein (eGFP), enabling luminescence-based quantification of live tumor cells. THP1-FG refers to THP1 cells engineered to overexpress the FG reporter. THP1-D-FG indicates FG-expressing THP1 cells treated with decitabine, while THP1-F-FG denotes FG-expressing THP1 cells treated with fludarabine.

Representative of 3 experiments. Data are presented as the mean ± SEM. ns, not significant, $*p < 0.05$, $**p < 0.01$, $***p < 0.001$, $****p < 0.0001$, by one-way ANOVA.

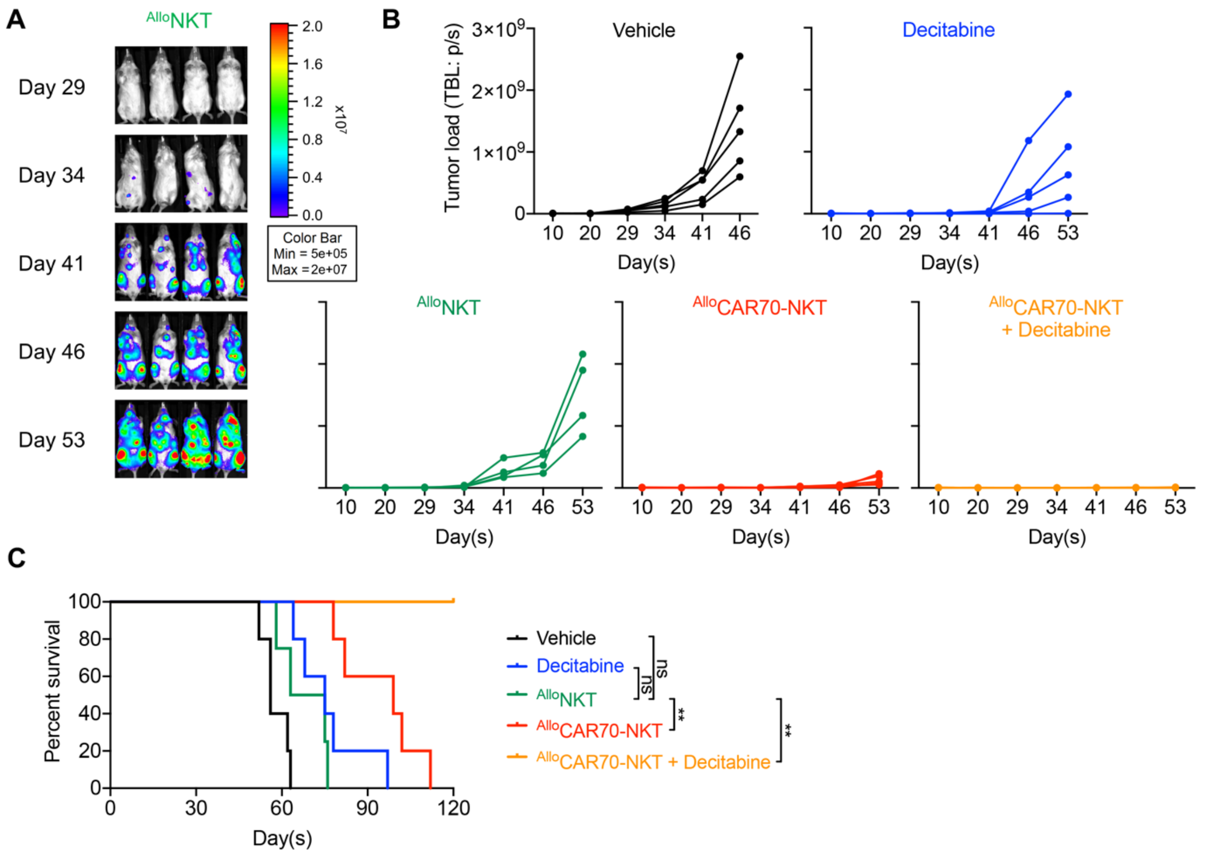

Figure S5. Studying the *in vivo* antitumor efficacy of ^{Allo}NKT and ^{Allo}CAR70-NKT cells using a THP1-FG human AML xenograft model.

(A) BLI images showing the presence of THP1-FG tumor cells in experimental mice over time.

(B) Quantification of (A) (n = 4 or 5). The data for Vehicle, Decitabine, ^{Allo}CAR70-NKT, and ^{Allo}CAR70-NKT + Decitabine are presented in the main Figure 4C. Here, we show the individual datasets separately.

(C) Kaplan–Meier survival curves (n = 4 or 5). The data for Vehicle, Decitabine, ^{Allo}CAR70-NKT, and ^{Allo}CAR70-NKT + Decitabine are presented in the main Figure 4D.

Representative of 3 experiments. Data are presented as the mean ± SEM. ns, not significant, **$p < 0.01$, by log rank (Mantel-Cox) test adjusted for multiple comparisons.

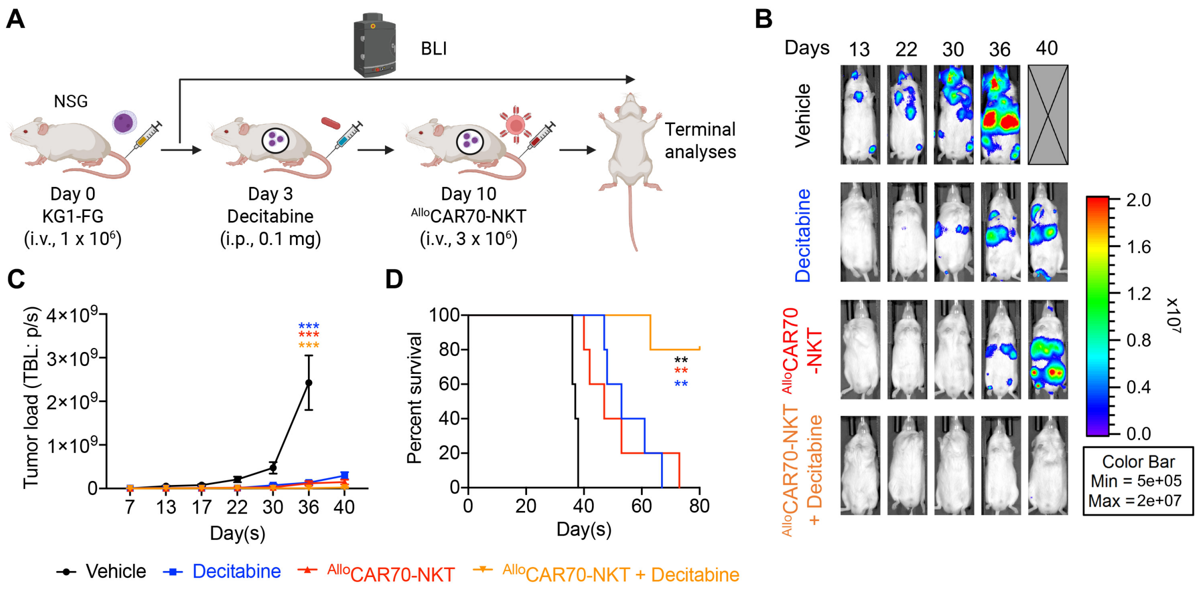

Figure S6. Synergistic effect of AlloCAR70-NKT cells with HMA in the treatment of CD70-negative AML using a KG1-FG xenograft mouse model.

(A) Experimental design to study the synergistic effect of AlloCAR70-NKT cells with Decitabine *in vivo*. A human CD70^{low} AML cell line KG1-FG was utilized.

(B) BLI images showing the presence of KG1-FG tumor cells in experimental mice over time.

(C) Quantification of (B) (n = 5).

(D) Kaplan–Meier survival curves (n = 5).

Representative of 2 experiments. Data are presented as the mean ± SEM. **p < 0.01, ***p < 0.001, by one-way ANOVA (C), or log rank (Mantel-Cox) test adjusted for multiple comparisons (D).

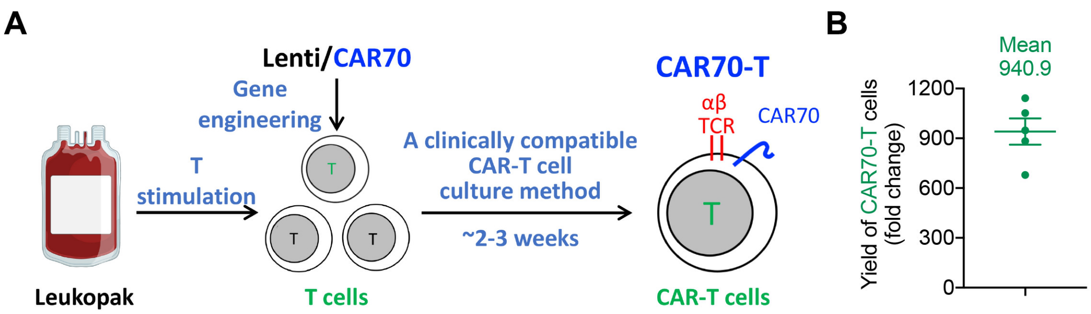

Figure S7. Generation of healthy donow PBMC-derived CAR70-engineered conventional T (CAR70-T) cells.

(A) Diagram showing the generation of CAR70-T cells.

(B) Yield of CAR70-T cells (n = 5).

Representative of 5 experiments. Data are presented as the mean ± SEM.

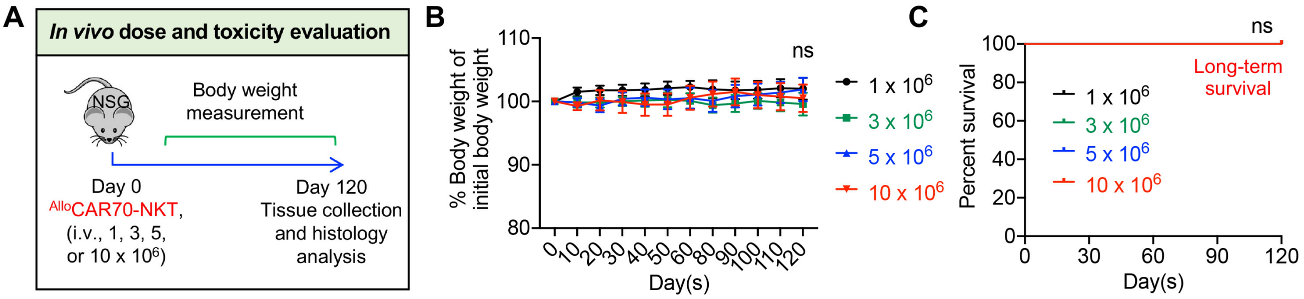

D

Group	Mouse ID	Pathologist's score																										
		Bone marrow			Spleen			Ileum			Pancreas			Kidney			Lung			Liver			Heart			Brain		
		Inf	HN	NHN	Inf	HN	NHN	Inf	HN	NHN	Inf	HN	NHN	Inf	HN	NHN	Inf	HN	NHN	Inf	HN	NHN	Inf	HN	NHN	Inf	HN	NHN
AlloCAR70-NKT	1	0	0	0	0	0	0	0	0	0	0	0	0	0	0	0	0	0	0	0	0	1	0	0	0	0	0	0
	2	0	0	0	0	0	0	0	0	0	0	0	0	0	0	0	0	0	0	0	0	0	0	0	0	0	0	0
	3	0	0	0	0	1	0	0	0	0	0	0	1	0	0	0	0	0	0	0	1	0	0	0	0	0	0	0
	4	0	0	0	0	0	0	0	0	0	0	0	0	0	0	0	0	0	0	0	0	0	0	0	0	0	0	0
	5	0	0	0	0	0	0	0	0	0	0	0	0	0	0	0	0	0	0	0	0	0	0	0	0	0	0	0
	6	0	0	0	0	0	0	0	0	0	0	0	0	0	0	0	0	0	0	0	0	0	0	0	0	0	0	0
Blank NSG	7	0	0	0	0	0	0	0	0	0	0	0	0	0	0	0	0	0	0	0	0	0	0	0	0	0	0	0
	8	0	0	0	0	0	0	0	0	0	0	0	0	0	0	0	0	0	0	0	0	0	0	0	0	0	0	0
	9	0	0	0	0	0	0	0	0	0	0	0	0	0	0	0	0	0	0	0	0	0	0	0	0	0	0	0
	10	0	0	0	0	0	0	1	0	0	0	0	0	0	0	0	0	0	0	0	0	0	0	0	0	0	0	0
	11	0	0	0	0	0	0	0	0	0	0	0	0	0	0	0	0	0	0	1	0	0	0	0	0	0	0	0
	12	0	0	0	0	0	0	0	0	0	0	0	0	0	0	0	0	0	0	0	0	0	0	0	0	0	0	0

Pathologist's score: 0 No abnormal findings; 1 Mild; 2 Moderate; 3 Severe

LEGEND: Inf: Inflammation; HN: Hematopoietic Neoplasm; NHN: Non-Hematopoietic Neoplasm

Figure S8. Studying the dose escalation and long-term toxicity of AlloCAR70-NKT cells using a human xenograft NSG mouse model.

(A) Experimental design.

(B) Body weight measured over time (n = 6).

(C) Kaplan–Meier survival curves (n = 6).

(D) Table summarizing the histopathological damage scores of the indicated organs collected from experimental mice that received 1 x 10^7 AlloCAR70-NKT cells or control (Blank NSG) on day 120.

Representative of 1 (D) and 3 (A-C) experiments. Data are presented as the mean ± SEM. ns, not significant, *p < 0.05, **p < 0.01, ***p < 0.001, ****p < 0.0001, by two-way ANOVA (B), or log rank (Mantel-Cox) test adjusted for multiple comparisons (C).
