# Supplementary material for: Synergizing hypomethylating agents with off-the-shelf CD70-targeted chimeric antigen receptor-engineered natural killer T cells for the treatment of acute myeloid leukemia
Source: Leukemia. 2026 Mar 26;40(5):880–93. doi: 10.1038/s41375-026-02930-5 (PMC13149322; doi:10.1038/s41375-026-02930-5)
Supplement: Supplementary file 2 — Supplementary methods [file 41375_2026_2930_MOESM2_ESM.pdf]

Supplementary methods

Synergizing hypomethylating agents with off-the-shelf CD70-targeted chimeric antigen receptor-engineered natural killer T cells for the treatment of acute myeloid leukemia

Yan-Ruide Li[1,2], Xinyuan Shen[1,2], Yuning Chen[1,2], Yichen Zhu[1,2], Jie Huang[1,2], Caspian Oliai[3], Lili Yang[1,2,4,5,6,7,8,§]

Author Affiliation:

[1]Department of Microbiology, Immunology & Molecular Genetics, University of California, Los Angeles, Los Angeles, CA 90095, USA

[2]Department of Bioengineering, University of California, Los Angeles, Los Angeles, CA 90095, USA

[3]Division of Hematology-Oncology, Department of Medicine, David Geffen School of Medicine, University of California, Los Angeles, Los Angeles, CA 90095, USA

[4]Jonsson Comprehensive Cancer Center, David Geffen School of Medicine, University of California, Los Angeles, Los Angeles, CA 90095, USA

[5]Eli and Edythe Broad Center of Regenerative Medicine and Stem Cell Research, University of California, Los Angeles, Los Angeles, CA 90095, USA

[6]Molecular Biology Institute, University of California, Los Angeles, CA 90095, USA

[7]Parker Institute for Cancer Immunotherapy, University of California, Los Angeles, Los Angeles, CA 90095, USA

[8]Goodman-Luskin Microbiome Center, University of California, Los Angeles, Los Angeles, CA 90095, USA

[§]Corresponding author. Email: liliyang@ucla.edu

Address Correspondence to:

Lili Yang, Ph.D.

Department of Microbiology, Immunology & Molecular Genetics

University of California, Los Angeles

Los Angeles, CA 90095, USA.

Phone: 310-825-8609, Email: liliyang@ucla.edu

Media and reagents

The X-VIVO 15 Serum-Free Hematopoietic Cell Medium (cat. no. 04418Q) was purchased from Lonza. The StemSpan™ T Cell Generation Kit (cat. no. 09940), comprising the StemSpan™ SFEM II Medium (cat. no. 09605), the StemSpan™ Lymphoid Progenitor Expansion Supplement (cat. no. 09915), the StemSpan™ LPMS (cat. no. 09930), the StemSpan™ Lymphoid Progenitor Differentiation Coating Material (cat. no. 09925), and the ImmunoCult™ Human CD3/CD28/CD2 T Cell Activator (cat. no.10970), and MethoCult™H4330 MethycelluloseBased Medium (cat. no. 04330) were purchased from StemCell Technologies. The CTS™ OpTmizer™ T-Cell Expansion SFM (no phenol red, bottle format, cat. no. A3705001), the RPMI 1640 cell culture medium (cat. no. MT10040CV), and the DMEM cell culture medium (cat. no. MT10013CV) were purchased from Thermo Fisher Scientific. The CryoStor® Cell Cryopreservation Media CS10 (cat. no. C2874) and Iscove's Modified Dulbecco's Medium (cat. no. I3390) was purchased from MilliporeSigma. The C10 medium was made of RPMI 1640 cell culture medium, supplemented with FBS (10% vol/vol), P/S/G (1% vol/vol), MEM NEAA (1% vol/vol), HEPES (10 mM), Sodium Pyruvate (1 mM), Beta-Mercaptoethanol (β-ME) (50 μM), and Normocin (100 μg/ml). The homemade D10 medium was made of DMEM supplemented with FBS (10% vol/vol), P/S/G (1% vol/vol), and Normocin (100 μg/ml). The homemade R10 medium was made of RPMI supplemented with FBS (10% vol/vol), P/S/G (1% vol/vol), and Normocin (100 μg/ml).

α-Galactosyl ceramide (αGC, KRN7000, cat. no. 867000) was purchased from Avanti Polar Lipids. Recombinant human IL-2 (cat. no. 200-02), IL-3 (cat. no. 200-03), IL-7 (cat. no. 200-07), IL-15 (cat. no. 200-15), IL-21 (cat. no. 200-21), IFN-γ (cat. no. 300-02), Flt3 ligand (Flt3L, cat. no. 300-19), macrophage colony stimulating factor (M-CSF, cat. no. 300-25), stem cell factor (SCF, cat. no. 300-07), and thrombopoietin (TPO, cat. no. 300-18) were purchased from Peprotech. Fetal Bovine Serum (FBS, lot no. 2087050) were purchased from Gibco and β-ME (cat. no. 1610710) were purchased from Bio-Rad. Penicillin Streptomycin-Glutamine (P/S/G, cat. no. 10-378-016), MEM nonessential amino acids (NEAA, cat. no. 11-140-050), HEPES Buffer Solution (cat. no. 15630080), and Sodium Pyruvate (cat. no. 11360070) were

purchased from Gibco. Normocin was purchased from Invivogen (cat. no. NC9390718). Decitabine (Tocris Bioscience™, cat. no. 26-2410) was purchased from Fisher Scientific. Azacitidine (5-Azacytidine, cat. no. A2385) was purchased from MilliporeSigma. Fludarabine (cat. no. 3495/10) was purchased from R&D system.

Mice

NOD.Cg-Prkdc^{SCID} Il2rg^{tm1Wjl} /SzJ (NOD/SCID/IL-2Rγ$^{-/-}$, NSG) mice were maintained in the animal facilities of UCLA. 6-10 weeks old male or female mice were used for all experiments unless otherwise indicated. Sex was not considered in the study design and analysis, as no significant differences were observed in the human AML xenograft mouse models used. All animal experiments were approved by the Institutional Animal Care and Use Committee of UCLA. All mice were bred and maintained under specific pathogen-free conditions, and all experiments were conducted in accordance with the animal care and use regulations of the Division of Laboratory Animal Medicine at the UCLA. Experimental mice were randomly assigned to treatment groups to avoid statistically significant differences in the baseline tumor burden.

Antibodies and flow cytometry

Fluorochrome-conjugated antibodies specific for human CD45 (Clone HI30, PerCP, FITC or Pacific Blue-conjugated, 1:500, cat. no. 304025, 304005, or 304021, RRID: AB_893341, AB_314393, or AB_493654), TCR αβ (Clone IP26, Pacific Blue or PE-Cy7-conjugated, 1:25, cat. no. 306715 or 306719, RRID: AB_1953256 or AB_10640829), CD3 (Clone HIT3a, Pacific Blue, PE, or PE-Cy7-conjugated, 1:500, cat. no. 300330, 300308, or 300316, RRID: AB_10551436, AB_314044, or AB_314052), CD1d (Clone 51.1, PE-Cy7 or APC-conjugated, 1:50, cat. no. 350310 or 350308, RRID: AB_2562408 or AB_10642829), CD4 (Clone OKT4, PE-Cy7, PerCP, or FITC-conjugated, 1:500, cat. no. 317414, 317432, or, 317408, RRID: AB_571959, AB_2028494, or AB_571951), CD8 (Clone SK1, PE, APC-Cy7, or APC-conjugated, 1:300, cat. no. 980902, 344713, or 344721, RRID: AB_ 2616623, AB_2044005,

or AB_2075390), CD14 (Clone HCD14, PE, PerCP, or FITC-conjugated, 1:100, cat.no. 325606, 325632, or 325604, RRID: AB_830679, AB_2563328, or AB_830677), CD19 (Clone HIB19, APC-Cy7-conjugated, 1:200, cat. no. 302218, RRID: AB_314248), CD20 (Clone 2H7, APC-Cy7-conjugated, 1:200, cat. no. 302314, RRID: AB_314262), CD27 (Clone M-T271, APC or FITC-conjugated, 1:50, cat. no. 356410 or 356404, RRID: AB_2561957 or AB_2561788), CD34 (Clone 581, PE-conjugated, 1:500, cat. no. 555822, RRID: AB_396151), CD31 (Clone WM59, FITC-conjugated, 1:100, cat. no. 303104, RRID: AB_314330), CD56 (Clone QA18A21, APC-Cy7, APC, or PE-conjugated, 1:20, cat. no. 398813, 398805, or 398803, RRID: AB_3097438, AB_2894509, or AB_2820070), CD69 (Clone FN50, PE-Cy7 or PerCP-conjugated, 1:50, cat. no. 310911 or 310927, RRID: AB_ 314846 or AB_10696423), CD70 (Clone WM53, APC, PE-Cy7 or PE-conjugated, 1:50, cat. no. 355110, 355112 or 355104, RRID: AB_2562481, AB_2687254 or AB_2561431), CD107a (Clone H4A3, FITC or PE-conjugated, 1:100, cat. no. 328606 or 328608, RRID: AB_1186036 or AB_1186040), CD112 (Clone TX31, APC or PE-conjugated, 1:200, cat. no. 337411 or 337409, RRID: AB_2565729 or AB_2174163), CD155 (Clone SKII.4, PE-Cy7-conjugated, 1:250, cat. no. 337613, RRID: AB_2565746), CD11b (Clone ICRF44, APC or PE-conjugated, 1:500, cat. no. 301310 or 301306, RRID: AB_314162 or AB_314158), MICA/MICB (Clone 6D4, APC-conjugated, 1:25, cat. no. 320907, RRID: AB_493196), 4-1BBL (Clone 5F4, PE-conjugated, 1:500, cat. no. 311504, RRID: AB_314883), CD83 (Clone HB15e, APC-Cy7-conjugated, 1:500, cat. no. 305330, RRID: AB_2566393), CD86 (Clone IT2.2, APC-conjugated, 1:500, cat. no. 305412, RRID: AB_493231), PD-1 (Clone A17188A, FITC, APC, or PE-conjugated, 1:50, cat. no. 379205, 379207, or 379209, RRID: AB_2922605, AB_2922606, or AB_2922607), TIM-3 (Clone A18087E, PE or APC-conjugated, 1:50, cat. no. 364805 or 364803, RRID: AB_2922577 or AB_2910409), LAG-3 (Clone 11C3C65, FITC, APC-Cy7, or PE-conjugated, 1:50, cat. no. 369307, 369347, or 369305, RRID: AB_2629750, AB_2922585, or AB_2629591), NKG2D (clone 1D11, PE-Cy7-conjugated, 1:50, cat. no. 320811, RRID: AB_2133275), DNAM-1 (Clone 11A8, APC-conjugated, 1:50, cat. no. 338311, RRID: AB_2561951), NKp30 (Clone P30-15, APC-conjugated, 1:50, cat. no. 325209, RRID:

AB_2149450), NKp46 (Clone 9E2, FITC-conjugated, 1:50, cat. no. 331922, RRID: AB_2561965), IFN-γ (Clone B27, PE-Cy7-conjugated, 1:50, cat. no. 506517, RRID: AB_2123322), Granzyme B (Clone QA16A02, APC or FITC-conjugated, 1:5000, cat. no. 372204 or 372206, RRID: AB_2687028 or AB_2687030), Perforin (Clone dG9, PE-Cy7-conjugated, 1:50 or 1:100, cat. no. 308125, RRID: AB_ 2572048), TNF-α (Clone MAb11, FITC or APC-conjugated, 1:1000, cat. no. 502906 or 502912, RRID: AB_315258 or AB_315264), IL-2 (Clone MQ1-17H12, APC-Cy7-conjugated, 1:50, cat. no. 500341, RRID: AB_ 2562854), β2-microglobulin (B2M) (Clone 2M2, PE or FITC-conjugated, 1:2000 or 1:5000, cat. no. 316306 or 316304, RRID: AB_492839 or AB_492837), HLA-DR (Clone L243, APC-Cy7-conjugated, 1:200 or 1:500, cat. no. 307618, RRID: AB_493586), and HLA-DR, DP, DQ (Clone Tü39, APC or PE-conjugated, 1:250, cat. no. 361714 or 361716, RRID: AB_2750316 or AB_2750318) were purchased from BioLegend. Fluorochrome-conjugated antibodies specific for mouse CD11b (Clone M1/70, APC or FITC-conjugated, 1:200, cat. no. 101212 or 101206, RRID: AB_312795 or AB_312789) and F4/80 (Clone BM8, PE or FITC-conjugated, 1:100, cat. no. 123110 or 123108, RRID: AB_893486 or AB_893502) were purchased from BioLegend. Fluorochrome-conjugated antibodies specific for human iNKT TCR Vα24-Jβ18 (Clone 6B11, PE-conjugated, 1:20, cat. no. 552825, RRID: AB_394478) were purchased from BD Biosciences. Fluorochrome-conjugated antibodies specific for human fibroblast activation protein FAP (FAP; Clone 427819, PE-conjugated, 1:100, cat. no. FAB3715P-100, RRID: AB_3086725), ULBP-1 (Clone 170818, PE-conjugated or unconjugated, 1:25, cat. no. FAB1380P or MAB1380, RRID: AB_2687471 or AB_2214683), and ULBP-2,5,6 (Clone 165903, APC-conjugated, 1:25, cat. no. FAB1298A, RRID: AB_2257142) were purchased from R&D Systems. A goat anti-mouse IgG F(ab')2 secondary antibody was purchased from ThermoFisher (HRP-conjugated, 1:50, cat. no. 31436, RRID: 228313). Fixable Viability Dye eFluor506 (e506; 1:500, cat. no. 65-0866-18) was purchased from Affymetrix eBioscience; mouse Fc Block (anti-mouse CD16/32, Clone 2.4G2, 1:50, cat. no. 553141, RRID: AB_394656) was purchased from BD Biosciences; and human Fc Receptor Blocking Solution (TruStain FcX, 1:100, cat. no. 422302, RRID: AB_2818986) was purchased from BioLegend. In our

study, note the use of antibodies with identical clones but differing conjugated fluorochromes, with one typical antibody listed herein.

All flow cytometry staining was performed following standard protocols, as well as specific instructions provided by the manufacturer of a particular antibody. Appropriate isotype staining controls were used for all staining procedures. Stained cells were analyzed using a MACSQuant Analyzer 10 flow cytometer (Miltenyi Biotech), following the manufacturer's instructions. FlowJo software version 9 (BD Biosciences) was used for data analysis.

Enzyme-linked immunosorbent cytokine assays (ELISAs)

The ELISAs for measuring human cytokines were conducted according to a standard protocol provided by BD Biosciences. Supernatants from cell culture experiments were collected and analyzed to quantify cytokines (e.g., human IFN-γ, TNF-α, IL-4, IL-10, and IL-15). The capture and biotinylated antibodies used for cytokine detection were sourced from BD Biosciences, while the streptavidin-HRP conjugate was obtained from Invitrogen. Human cytokine standards were purchased from eBioscience, and the Tetramethylbenzidine (TMB) substrate was acquired from Thermo Scientific (cat. no. PI34021). Human IL-17A ELISA Kits were purchased from Invitrogen (cat. no. BMS2017). Absorbance of the samples was measured at 450 nm using an Infinite M1000 microplate reader (Tecan).

Histology analysis

Tissues were harvested from experimental mice, fixed in 10% neutral buffered formalin for up to 36 hours, and embedded in paraffin for sectioning (5 mm thickness). Tissue sections were subsequently prepared and stained with hematoxylin and eosin by the UCLA Translational Pathology Core Laboratory (TPCL) in accordance with the Core's standard protocols. Stained sections were imaged using an Olympus BX51 upright microscope equipped with an Optronics Macrofire CCD camera (AU Optronics), and the images were analyzed using an Optronics PictureFrame software (AU Optronics).

Statistics

Graphpad Prism 9 software (Graphpad) was used for statistical data analysis. Student's two-tailed *t* test was used for pairwise comparisons. Ordinary 1-way ANOVA followed by Tukey's or Dunnett's multiple comparisons test was used for multiple comparisons. Log rank (Mantel-Cox) test adjusted for multiple comparisons was used for Meier survival curves analysis. Data are presented as the mean ± SEM, unless otherwise indicated. In all figures and figure legends, "n" represents the number of samples or animals used in the indicated experiments. A P value of less than 0.05 was considered significant. ns, not significant.

In this study, the sample size was selected based on power calculations ensuring sufficient sensitivity to detect a predefined effect size with acceptable statistical confidence. For animal studies, the sample sizes were estimated based on prior experience with similar animal studies to ensure adequate biological variability and feasibility.

Animals were excluded from analysis only if they exhibited unexpected health issues unrelated to the study intervention, failed to engraft as confirmed by predefined criteria, or if sample collection was incomplete or technically compromised. Group assignment was randomized manually by an investigator not involved in downstream data collection, using baseline measurements to ensure even distribution across treatment arms.

The investigator was blinded to group allocation during outcome assessment, with samples or animals labeled by coded identifiers to prevent bias in data collection and analysis.
